# A Comprehensive Review of Common Bacterial, Parasitic and Viral Zoonoses at the Human-Animal Interface in Egypt

**DOI:** 10.3390/pathogens6030033

**Published:** 2017-07-21

**Authors:** Yosra A. Helmy, Hosny El-Adawy, Elsayed M. Abdelwhab

**Affiliations:** 1 Food Animal Health Research Program, Department of Veterinary Preventive Medicine, Ohio Agricultural Research and Development Center, The Ohio State University, Wooster, OH 44691, USA; 2 Department of Animal Hygiene, Zoonoses and Animal Ethology, Faculty of Veterinary Medicine, Suez Canal University, 41511 Ismailia, Egypt; 3 Friedrich-Loeffler-Institut, Federal Research Institute for Animal Health, Institute of Bacterial Infections and Zoonoses, Naumburger Str. 96a, 07743 Jena, Germany; 4 Faculty of Veterinary Medicine, Kafrelsheikh University, 335516 Kafrelsheikh, Egypt; 5 Friedrich-Loeffler-Institut, Federal Research Institute for Animal Health, Institute of Molecular Virology and Cell Biology, Suedufer 10, 17493 Greifswald-Insel Riems, Germany

**Keywords:** zoonotic diseases, Egypt, influenza, MERS-CoV, rabies, cryptosporidiosis, fascioliasis, campylobacteriosis, salmonellosis, Middle East

## Abstract

Egypt has a unique geographical location connecting the three old-world continents Africa, Asia and Europe. It is the country with the highest population density in the Middle East, Northern Africa and the Mediterranean basin. This review summarizes the prevalence, reservoirs, sources of human infection and control regimes of common bacterial, parasitic and viral zoonoses in animals and humans in Egypt. There is a gap of knowledge conerning the epidemiology of zoonotic diseases at the human-animal interface in different localities in Egypt. Some zoonotic agents are “exotic” for Egypt (e.g., MERS-CoV and Crimean-Congo hemorrhagic fever virus), others are endemic (e.g., Brucellosis, Schistosomiasis and Avian influenza). Transboundary transmission of emerging pathogens from and to Egypt occurred via different routes, mainly importation/exportation of apparently healthy animals or migratory birds. Control of the infectious agents and multidrug resistant bacteria in the veterinary sector is on the frontline for infection control in humans. The implementation of control programs significantly decreased the prevalence of some zoonoses, such as schistosomiasis and fascioliasis, in some localities within the country. Sustainable awareness, education and training targeting groups at high risk (veterinarians, farmers, abattoir workers, nurses, etc.) are important to lessen the burden of zoonotic diseases among Egyptians. There is an urgent need for collaborative surveillance and intervention plans for the control of these diseases in Egypt.

## 1. Introduction

Zoonotic diseases (ZD) are those infections that can be naturally transmitted from animals to humans with or without vector [1]. In the past few decades, there has been a rise in the outbreaks of zoonotic diseases which have an enormous socioeconomic impact worldwide, for instance, all foodborne zoonoses occured in a single country costs about $1.3 billion annually [2]. Additionally, ZD constitute 61% of all communicable diseases causing illness in humans and about 75% of emerging human pathogens [3,4,5]. More than 75% of the diseases that affected humans have been transmitted from animals or animal products [1]. Zoonotic diseases can be transferred from animals to humans by several ways such as the consumption of contaminated food and water (e.g., cryptosporidiosis), exposure to the pathogen during processing (e.g., campylobacteriosis and salmonellosis), direct contact with infected animals (e.g., avian influenza) and by pets scratches or bite (e.g., rabies) [6,7,8,9,10]. Some emerging zoonoses expanded their host range and their incidence increased (e.g., avian influenza). This expansion occurred as a result of global trade, increase poultry production, climate changes, bird migration, human movement, and the burgeoning global population. Several animal reservoirs of ZD have been identified, including ruminants, equines, poultry, rodents, dogs and cats, mosquitoes and ticks, which were considered a potential risk of disease transmission [11,12,13,14]. Furthermore, antimicrobial resistance considered as an additional risk associated with exposure to zoonotic pathogens because it is potentially limits disease treatment options in both public health and veterinary settings [14]. Therefore, prevention and control programs should be implemented by public health and veterinary officers to combat the sources and reservoirs of zoonoses especially after the development of antimicrobial resistance problem due to misuse of antibiotics [12]. 

Egypt is located in the north-eastern part of Africa connecting the three old-world continents Africa, Asia and Europe. It is the county with the highest population density in the Middle East, North Africa and the Mediterranean basin with about 90 million inhabitants. Egypt has 27 governorates and over 90% of the population live in 10% of the whole area along the River Nile and Nile Delta in the northern part of the country. A number of zoonotic pathogens have been reported in Egypt. The burden of ZD is provoked by factors such as the method of control, environmental factors, behavioral factors, social factors, clinical manifestations, the socioeconomic impact of such diseases and mode of transmission. The highest incidence and prevalence of zoonotic diseases in Egypt may be attributed to the deficiency of suitable control mechanisms, inadequate infrastructure and lack of information on their significance and distribution. However, there is a marked decrease in the prevalence of some ZD (e.g., schistosomiasis). In this review, we will focus on the most important and prevalent emerging and re-emerging ZD in Egypt including the current situation, reservoirs, sources of human infection and control regimes, if available.

## 2. Bacterial Zoonosis

### 2.1. Campylobacteriosis

Campylobacteriosis is a zoonotic disease which has a worldwide public health impact and is caused mostly by *Campylobacter jejuni* or *Campylobacter coli* [15]. *Campylobacter* is S-shaped, or curved rod-shaped bacteria of the epsilon class of Proteobacteria [16]. Poultry are a natural reservoir and are frequently colonized with thermophilic *Campylobacter* species, primarily *C. jejuni* and *C. coli*. Although *Campylobacter* is insignificant for poultry health, it is a leading cause of foodborne gastroenteritis in humans worldwide. Contaminated poultry carcasses are recognized as the main source for human exposure [17]. It is extremely difficult to keep poultry flocks free of *Campylobacter* which is commonly present in the poultry houses environment. During the slaughtering process, contamination of the carcasses with high numbers of *Campylobacter* is unavoidable [18]. 

The disease is endemic in Egypt and is a major cause for pediatric diarrhea. Nonetheless, the epidemiology in animals and humans has not been fully characterized. In the period from 2006 to 2015, several studies described the isolation of *Campylobacter*, mainly *C. jejuni*, in chickens, raw milk, milk products, diarrheic and normal camel calves and stool of diarrheic patients in several governorates in Egypt [19,20,21,22,23]. Backyard poultry remains the main source of *Campylobacter* transmission to humans [24,25,26]. In one governorate, *Campylobacter* spp. including *C. jejuni* and *C. coli* were isolated from 47.5% of chicken and 2.7% of human samples [27]. Chickens and humans isolates were genetically similar signifying the high possibilities of zoonotic transmission from animals to humans [20,28]. Isolation of *Campylobacter* from diarrheic children was more frequent (17.2%) than *Salmonella* (3%), *Shigella* (2%), or other bacterial pathogens (1%) [29]. In another governorate, 59 *C. jejuni* and/or *C. coli* isolates were recovered from diarrheic humans. The isolates belonged to 14 groups for *fla*A and 11 groups for *fla*B genes indicating considerable genetic variability among isolates belonging to the same serogroup [30]. 

### 2.2. Salmonellosis

Salmonellosis is a very common zoonotic infection which may be mild or severe life-threatening disease. Salmonella, the causative agent, is gram-negative bacilli belonging to the family *Enterobacteriaceae* and has 2 species: *Salmonella bongori* and *Salmonella enterica*. The latter has over 2500 different serotypes or serovars. *Salmonella enterica* serovar Enteritidis is the major cause of human salmonellosis outbreaks in the United States and Europe [31,32]. Live animals and consumption of contaminated food of animal origin (e.g., eggs, meat, poultry and milk) have been implicated in transmission of the bacterium to humans. Also, human-to-human transmission through the fecal-oral route is possible [33,34]. 

*Salmonella enteritidis* was isolated from chickens and different animals in Egypt [33]. Salmonella infection in poultry including commercial broilers in northern Egypt during 2014–2015 [35], in eastern Egypt during the period of December 2009 to May 2010 [36] and from different markets in south of Egypt were described [37]. A highly prevalence of eggshell contamination in laying hen flocks infected with *Salmonella enteritidis* was reported [38]. In Cairo, surveillance in pigeons indicated the spread of *Salmonella* serotype Typhimurium, Braenderup and Lomita which also endanger the public health [39]. Moreover, *Salmonella* spp. was recovered from diarrheic neonatal calves [40]. Infections among imported ducklings and camels were considered as an additional source for salmonellosis in Egypt [41,42]. 

Furthermore, *Salmonella enterica* was isolated from food samples collected from different street venders, butchers, retail markets and slaughterhouses in Egypt [43,44]. A study described the prevalence of *Salmonella* species in dairy handlers as well as milk and dairy products randomly collected from different dairy farms and supermarkets. Two stool specimens out of 40 apparently healthy dairy handlers were positive by PCR [45]. *Salmonella* was also detected in 9.8% of the 225 seafood samples tested [46]. It is worth mentioning that typhoid fever is endemic in Egypt; and quinolones are the empirical treatment of choice. There are limited data reporting quinolone resistance among Egyptian typhoidal *Salmonella* isolates [47,48]. However, 68% of *Salmonella enterica* isolates showed multidrug resistance phenotypes [43] particularly against chloramphenicol and trimethoprim-sulfamethoxazole, streptomycin, tetracycline, ampicillin and gentamicin [49] which is of great health significance [43]. Numerous genes linked to antimicrobial resistance of *Salmonella* were prevalent in examined isolates from chicken and human origin in Egypt [49]. 

### 2.3. Brucellosis

Brucellosis remains one of the major zoonotic infections worldwide [50]. The disease is caused by a gram-negative coccobacillus bacterium of the genus Brucella, firstly discovered in 1887 by David Bruce. Since then, a number of species have been identified: for instance *B. abortus* in cattle, *B. melitensis* in sheep and goats and *B. suis* in pigs [51,52]. In animals, brucellosis causes economic losses because of abortions, decreased milk production, sterility, and veterinary care and treatment costs [51]. In humans, infection can be long lasting and is accompanied by flu-like illness with or without neurological and genitourinary complications. Zoonotic infection occurs after the contact with animals or animal products contaminated with the bacteria. Although brucellosis is a neglected disease in developed countries, it remains a major challenge for animal and human health in developing countries particularly in the Mediterranean basin, Asia and Latin America [53]. 

In Egypt, brucellosis was first described in 1939 [53]. Mass vaccination of animals and test-and-slaughter of serologically positive animals are the main control tools costing over $3 million annually [54]. Nevertheless, Brucella became endemic. There are limited data on the prevalence of brucellosis in animals and humans in Egypt [54]. The infection of sheep and goats by *B. melitensis* and cattle and buffaloes by *B. abortus* has been frequently reported [53,54]. In 2016, the prevalence of animal and human brucellosis was 7% and 1.25%, respectively, at a district located in the Nile Delta with high livestock density [55]. In another serosurvey in East of Egypt, 4.42% and 8.91% in private farms and individual cases, respectively, were found positive. *Brucella melitensis* biovar 3 was isolated from seroreactive animals [56]. In the period between 2006 and 2008, the prevalence of brucellosis among sheep, goats and cattle flocks in 18 Egyptian governorates were 26.7%, 18.9% and 17.2%, respectively [57]. In humans, up to 70 persons per 100,000 population were found positive to *Brucella* in 2002 and 2003; 70% were male with a median age of 26 years. The infection was associated with close contact to animals and consumption of unpasteurized milk products [58].

### 2.4. Escherichia coli

*Escherichia coli* is gram-negative facultative bacteria that belongs to the family *Enterobacteriaceae*. Although it is normally commensal in nature and animals, many strains are food- and waterborne zoonotic pathogens. Some strains like O157 and other enterohemorrhagic *E. coli* (EHEC) cause no discernible disease in their animal reservoirs; however, diarrhea, hemorrhagic colitis, and hemolytic uremic syndrome are not uncommon in humans [59]. 

In Egypt, many studies have shown a high prevalence of *E. coli* O157 strains in dairy and meat products in different locations [44,60,61]. Avian pathogenic *Escherichia coli* (APEC) infection is responsible for great economic losses in poultry industry. Avian *E. coli* strains from broiler flocks in Egypt were genetically similar to *E. coli* associated with human infection [62] with prevalence rate in chicken visceral and human stool samples of 26.9% and 46.2%, respectively [63]. Enteropathogenic *E. coli* (EPEC), diffuse-adhering *E. coli* (DAEC) and enteroaggregative *E. coli* (EAEC) are associated with infantile diarrhea in Egyptian children where 220 enteroadherent *E. coli* were identified from 729 diarrheic children [64]. In another study, *E. coli* was isolated from 52.1% hospitalized patients and 60.4% from outpatient clinics with high degrees of resistance to ciprofloxacin and co-trimoxazole [65]. Also, there is increasing incidence of carbapenemase- and extended-spectrum β-lactamase-producing *Enterobacteriaceae* in dairy farms and hospital-acquired infections. This represents a major health problem because of the few therapeutic alternatives [66].

### 2.5. Listeriosis

Listeriosis is one of the foodborne pathogens with high fatality rate. It is caused by *Listeria monocytogenes*, a gram-positive bacterium which is able to grow at temperatures as low as 0 °C challenging the control in human foodstuffs. *L. monocytogenes* can persist for long periods in the environment or as an asymptomatic infection in adult animals and birds. Neurological disorders due to encephalitis are the most common clinical signs in ruminants, in addition to late abortion. Consumption of raw milk, cheeses, raw-meat products, poultry and fish is the major sources of infection. Human listeriosis is associated with serious invasive illness, particularly in elderly and immunocompromised patients, pregnant women, newborns and infants [67].

In Egypt, existence of the organism in water [68,69], contaminated food [70], seafood [71], milk products and milk samples from apparently healthy buffaloes, cows, she-camels, goats and sheep has been reported [72,73,74,75]. During 2013, *Listeria* spp. was detected in 47.5% of 200 poultry farm samples in Egypt [76]. The isolation rates of *L. monocytogenes* from different localities in Egypt were 8% in beef burger, 4% in minced meat and 4% in luncheon meat; while sausage samples were all negative [77]. In humans, *L. monocytogenes* is considered the most common lethal complication in Egyptian patients with liver cirrhosis associated with ascites [78]. Detection of the organism in stool samples of hospitalized Egyptians was reported in different localities [71,79]. 

### 2.6. Q Fever

Q fever is a zoonotic bacterial disease with public health implications. The disease is caused by *Coxiella burnetii*, a gram- negative bacteriumthat mostly affect ruminants. The bacterium causes abortion in sheep, goat and cattle and is excreted in infected animal feces, urine, milk, and birth products. People can get infected by inhalation of contaminated materials. *C. burnetii* causes febrile flu-like illness and pneumonia in poor hygiene settings. The epidemiology of Q fever in Africa is poorly understood [80,81]. 

The epidemiology of *C. burnetii* in Egypt is not well-known [82]. Antibodies were detected in Egyptian donkeys, goats, sheep, pigs, dogs and rats [83]. Q fever may be enzootic in cattle in northern Egypt [84]. In 2006 and 2011, *C. burnetii* antibodies were detected in up to 32.7%, 23.3%, 13.3% and 13% of sheep, goats, camels and cattle samples in different localities in Egypt, respectively [82,85,86]. However, all seropositive animals were negative for *C. burnetii* DNA by PCR [85]. In 2009, the prevalence of *C. burnetii* in blood samples collected from domestic and imported livestock slaughtered at the abattoir in central Egypt was 4% in buffalo, 8% in sheep, and 70% in camels [87]. The prevalence of antibodies to *C. burnetii* among Egyptians in various locations was 5 to 28% particularly among cattle workers, veterinarians and veterinary assistants and those live in agricultural districts [82,88,89].

## 3. Parasitic Zoonosis

### 3.1. Schistosomiasis

Schistosomiasis is the second most common parasitic infection globally after malaria [90] and considered as one of the Neglected Tropical Diseases (NTDs) [91]. Schistosomiasis was reported in more than 200 million people in 74 countries and nearly 800 million people are at risk of infection worldwide [90,92]. Humans acquire the infection by contact with or drinking of contaminated water. There are three main species infecting humans including *S. mansoni*, *S. haematobium* and *S. japonicum* complex (*S. japonicum* and *S. mekongi*) [93]. Infection with *Schistosoma* spp. results in skin rash or itching, flu-like illness and severe intestinal and urinary tract disorders [94,95]. Chronic schistosomiasis can persist for years and lead to neurological complications and death [96,97]. 

Schistosomiasis is one of the most endemic parasitic diseases in Egypt. The earliest case of human schistosomiasis (*S. haematobium*) occurred more than 5000 years ago in an Egyptian adolescent [98]. In addition, *S. haematobium* calcified eggs were identified in two mummies aged 3000 and 4000 years old of the 20th Dynasty [99,100]. Between 1935 and 2010, the infection rate was very high in school-age children and the infection can persist among adults [95,101,102]. Furthermore, working in agriculture is a risk factor for *Schistosoma* spp. infection [103]. The prevalence of *S. haematobium* and *S. mansoni* in Egypt ranged between 0.08% and 75% [104,105,106,107,108,109,110,111,112,113,114,115,116,117]. The highest prevalences of *S. haematobium* were detected in lower Egypt [106,109], while the highest prevelance of *S. mansoni* was detected around central Egypt [102,118,119]. The overall prevalence of schistosomiasis in Egypt declined year by year to reach 10% in 1999, 5% in 2000, 3.5% in 2002, 1.2% in 2006 and 3–10% in 2010 [102,120,121,122]. This continuous decrease was due to the control of schistosomiasis by using praziquantel in mass chemotherapy and due to snail control by using anti-bilharzial chemotherapy [102,123,124].

### 3.2. Fascioliasis

Fascioliasis is caused by a liver fluke belonging to genus *Fasciola* (*Fasciola hepatica* and *F. gigantica*). *Fasciola* is one of the neglected foodborne trematodes that infect ruminants and humans worldwide [125,126,127]. It was estimated that fascioliasis, associated with other diseases, especially schistosomiasis, affects at least 2.4 million people in more than 70 countries [128,129,130]. It causes gastrointestinal problems and chronic fascioliasis results in jaundice and inflammation of the liver, gallbladder and pancreas [129]. Fecal excretion of eggs from infected animals (e.g., cattle, sheep, buffaloes, donkeys and pigs) in fresh-water is the main source of infection. After hatching, larva lodge in a particular type of water snail (the intermediate host). Carrier animals are infected by eating metacercaria encysted on leaves of water plants or vegetables [127].

Egypt is one of the endemic areas with fascioliasis in the world [131] with annual loss in milk and meat being estimated to be 30% [132]. The climatic factors influence the incidence of fascioliasis and all snail transmitted parasites in Egypt [128,133]. *F. gigantica* is considered the endogenous fasciolid species in Egypt with tropical and subtropical distribution [134] while *F. hepatica* originated from Europe and introduced to Egypt through the importation of domestic animals [135,136]. *Fasciola* spp. infected different animal species in Egypt including; sheep, goats, cattle, buffaloes, horses, donkeys, camels and rabbits and the infection rates reach 90% in some areas [137,138]. The overall prevalence in Egypt is unknown because reports show wide variations in infection rates [139]. Between 1959 and 2016, the prevalence of *Fasciola* spp. among different animal species in different localities in Egypt ranged between 0% and 59% [138,139,140,141,142,143,144,145,146]. In 1988, fascioliasis has been reported in approximately all Egyptian governorate where up to 60% of cattle and buffaloes and 78% of sheep were positive [139]. 

Since 1980, human fascioliasis was reported in most of the Egyptian governorates especially in the Nile Delta [130,139]. It was estimated that 830,000 humans are infected and 27 million of persons are exposed to the risk of infection in Egypt [147]. Between 1958 and 2006, fascioliasis has been reported in humans in different localities in Egypt and the prevalence rate was between 2% and 19% [130,139,148,149,150,151,152,153]. From 1998 to 2002, the prevalence of fascioliasis in the treated endemic areas was reduced from 5.6 to 1.2% [154]. This decrease in the prevalence was continued in 2009 to reach 0% [142]. However, in 2013, the prevalence increased again to reach 8% [155,156]. Triclabendazole is the selective treatment of fascioliasis in specific high-risk age groups such as school children and villagers [154].

### 3.3. Cryptosporidiosis

Cryptosporidiosis is a zoonotic protozoal disease caused by the genus *Cryptosporidium* [157]. *Cryptosporidium* spp. was discovered by Tyzzer in mice [158] and the first human case was reported in 1976 [159]. Afterwards, more attention was given to cryptosporidiosis since it was determined to cause death in one acquired immune deficiency syndrome (AIDS) patient [159]. Cryptosporidia essentially entered veterinary medicine only in the early 1980s with reports of cryptosporidium-associated neonatal calf diarrhea [160] and established as a primary enteric pathogen [161]. The importance of *Cryptosporidium* as a public health problem began in 1993, when more than 400,000 residents in Milwaukee, WI, USA were affected by *C. hominis* due to the consumption of contaminated drinking water. This was reported as the largest world waterborne outbreak [162,163,164]. Out of 26 *Cryptosporidium* species, *C. hominis* and C. parvum are responsible for more than 90% of human cases of cryptosporidiosis [165], exhibiting acute self-limiting diarrhea in immunocompetent persons and life-threatening diarrhea in immunocompromised persons [166]. It was estimated that 1 to 10% of the developing countries’ populations were infected with *Cryptosporidium* particularly among 1-to-9-year-old children and toddlers [167]. 

Reports on cryptosporidiosis in Egypt are rare. The highest prevalence rates were reported in rural areas where there is close contact with animals. The accuracy of the reported prevalence rate depends on the used detection method [168]. In animals, from 1999 to 2016 the prevalence rate of *Cryptosporidium* infection ranged between 2% and 69% among different species including cattle, buffalo calves, camels, sheep, goats, lambs, dogs, wild rats and quails. The highest prevalence rates were reported in governorates that located close to the River Nile [169,170,171,172,173,174,175,176,177,178,179,180,181]. In humans, between 1989 and 2016 *Cryptosporidium* infection has been reported in almost all Egyptian governorates with prevalence rates ranged between 3% and 50% or up to 91% in immunocompromised patients and diarrheic children [169,170,171,181,182,183,184,185,186,187,188,189,190,191,192,193,194,195,196,197]. 

### 3.4. Giardiasis

*Giardiasis* is caused by *Giardia duodenalis* (syn. *G. intestinalis*, *G. lamblia*) and considered one of the most common intestinal protozoal parasites affecting humans worldwide [198]. It also infects other mammals, including pets and livestock [199]. The high-risk group is children, especially those in daycare settings, orphanages and primary schools [200] with 2.5 million annual cases in the developing countries [201]. Infection with *Giardia* spp. results in gastrointestinal disorders [200]. *Giardia* cysts can be transmitted to humans through ingestion of contaminated water or food, but the parasite can be transmitted directly from infected individuals [202]. *G. duodenalis* has been divided into eight different assemblages (A–H) that have varied host specificities [203]. Assemblages A and B have been found to infect humans and other mammals [200]. 

In Egypt, few studies have been done to identify the relation between different assemblages and the presence of symptoms, especially among children and animals [204,205]. Between 2004 and 2016, the prevalence of *Giardia* spp. ranged between 2% and 53% among different animal species including ruminants, dairy cattle, stray cats, wild rats and fish [152,204,206,207,208]. Assemblage E was also more prevalent among ruminants and dairy cattle [204,206]. In humans, between 2001 and 2017, Giardiasis has been reported in different localities of Egypt and the prevalence rates ranged between 10% and 75% [152,192,204,206,209,210,211,212,213,214,215,216]. The most prevalent *G. duodenalis* genotypes were assemblage A and B and to lesser extent assemblage E among diarrheic patients in Egypt [204,205,206,209,214,215,217,218,219,220]. The prevalence of *G. duodenalis* was high in rural areas more than in urban areas which was attributed to the higher exposure to multiple risk factors such as poor water supply, poor sanitation, and presence of animals [204]. Interestingly, asymptomatic Giardia infection can persist for 4 months with 4.5 episodes per child/year in rural areas in Egypt [213].

### 3.5. Toxoplasmosis

*Toxoplasma gondii* (*T. gondii*) is one of the most important human zoonotic protozoan parasites, infecting one third of the world’s population [221]. Infection with *T. gondii* is prevalent in humans and animals, including poultry [222]. Cats are considered the key host in the transmission of *T. gondii* to humans and other animals as they excrete the environmentally resistant oocysts in their feces [223]. Human infections occur through ingestion of food or water containing viable cysts [224], congenitally, by blood transfusions or organs transplantation [225]. The high risk groups are immunocompromised patients and fetuses whose mothers acquire acute infection during pregnancy [226]. Infection with *T. gondii* is asymptomatic in immunocompetent and primary infected pregnant women; however, severe complication and death have been also reported [224,226,227,228].

In Egypt, indoor and outdoor cats are allowed to roam, where they hunt their own food or live on scraps of garbage. Therefore, the environment is highly contaminated with oocysts excreted by these cats. This might affect livestock that will later be slaughtered for human consumption [229]. In 1980s, seroprevalence of the parasite ranged between 16% and 59% in stray and domestic cats [230,231,232] while in 2008, antibodies were reported in 57% of cats [233]. Recently, seroprevalence of *T. gondii* was up to 98%, indicating high environmental contamination with oocysts [207,229,234]. Furthermore, *T. gondii* antibodies were detected in 10% to 62% of ruminants, including cattle, sheep and goat and in equines, including horses and donkey [235,236,237,238,239,240]. Between 1990 and 2016, seroprevalence of *T. gondii* in chickens, turkeys and ducks ranged between 9% and 85% in different localities in Egypt [241,242,243,244,245,246]. Therefore, consumption of undercooked poultry meat may be considered a risk factor for toxoplamosis in humans or animals [222]. In humans, there are limited data on the prevalence of *T. gondii* and associated risk factors in women during pregnancy. In different Egyptian localities, between 1993 and 2016, the prevalence of toxoplasma antibodies were ranged between 27% and 68% in pregnant women [236,239,245,247,248,249,250,251,252,253], 26% in cerebrospinal fluid of meningoencephalitis patients [254], 59.6% in asymptomatic blood donors [255]. 

## 4. Viral Zoonosis

### 4.1. Influenza

Influenza A viruses (IAV), members of the RNA family *Orthomyxoviridae*, have up to 144 subtypes according to the variation/combination of the surface glycoproteins hemagglutinin and neuraminidase. IAV are further classified to human influenza, swine influenza (SIV), bat influenza, equine influenza and avian influenza viruses (AIV). SIV and AIV transmit from swine or birds to humans, respectively, mostly via direct contact with infected animals. The infection in humans ranges from mild self-limiting respiratory-like illness to death [256,257]. 

Due to the very low pork production in Egypt, swine influenza is not a major ZD, although serosurveillance indicated infections of humans in 1979–1980 [258]. On the contrary, AIV are very important zoonotic viruses in Egypt. Poultry industry in Egypt was estimated in 2006 to be one billion birds with several millions of labors. In late 2005, the Asian highly pathogenic (HP) AIV of subtype H5N1 was firstly detected in wild migratory birds in a northern Egyptian wetland. In February 2006, the virus transmitted to domestic commercial and backyard birds and in March the first human case was recorded. To date, the virus is endemic in Egyptian birds causing tremendous economic impact despite the mass vaccination intervention strategy [259]. Another AIV subtype is H9N2 which, in poultry, did not cause severe illness unless the infection is complicated by secondary bacterial infection or immunosuppression. The endemic H9N2 in Egyptian poultry was firstly detected in 2011 and vaccination is widely used to control the infection [259].

In humans, Egypt is the country with the highest recorded cases worldwide. The fatality rate in Egypt is lower than the global rate. Thus, lower virulence and subsequent adaptation of the virus in human has been assumed. Many mutations that enhance virus binding to mammalian type receptors have been studied. Subclinical infection in human has been reported as revealed by serological surveillance [258,259]. Nevertheless, a recent study has shown that the virus has not yet acquired the aerogenic transmissibility as naïve ferrets cohoused with inoculated ferrets did not acquire infection [260]. The Egyptian H5N1 viruses are highly susceptible to antiviral drugs (Oseltamivir), but are resistant to amantadine. Infection is usually acquired by intensive contact particularly with backyard birds. Women and children are mostly affected. To date, there are 3 human infections by H9N2 viruses reported to the WHO [261]. Subclinical infection in poultry workers and co-infection of poultry and human with H5 and H9 in Egypt have been reported. Moreover, serosurvey revealed the presence of antibodies against H7 viruses in poultry [262] and in humans [263], but no virus was isolated, so far [262,263].

### 4.2. Rabies

The rabies virus (RABV) belongs to the genus Lyssavirus of the RNA family *Rhabdoviridae* within the order *Mononegavirales*. Rabies is a widespread neurological zoonotic disease of all warm-blooded animals including humans. Dogs are the most important host for RABV, however wild carnivores (e.g., fox) and bats are considered reservoirs. Infection in humans occurs by direct contact with mucosal surfaces (e.g., bites) or possibly through contamination of wounds with infected materials (e.g., scratches). The virus has a relatively long incubation period (according to the site of bite) which may reach a few months to a year giving the opportunity for immediate vaccination/treatment. Each year, about 60,000 fatal human infections are recorded worldwide [264,265].

In Egypt, RABV was recognized before 2300 B.C. [266]. Although the notifiable disease is now endemic in many regions throughout the country, there are no recent reported outbreaks of RABV. It is worth mentioning that the canine population in Egypt was estimated to exceed three million stray dogs and half a million owned dogs. Vaccination of dogs in Egypt was usually done by low-egg-passage of the Flury strain, which has now been replaced by attenuated tissue culture vaccines [266]. The disease transmitted to humans in Egypt mainly by dog bites, however the virus has been detected in other animals: cats, ruminants (e.g., buffaloes), horses, donkeys, rodents and mongooses [266,267,268,269]. In 1970s, the virus was isolated from several rodents and wild mammals including Gerbils, foxes and cats [270,271]. In 1988, Naval Medical Research Unit Three (NAMRU-3 is a biomedical research laboratory of the US Navy located in Cairo, Arab Republic of Egypt) isolated street RABV from dogs (*n* = 9), cats (*n* = 2), farm animals (*n* = 2), Gerbils (*n* = 3) and Jackal (*n* = 1). In 1990, an outbreak was reported to the OIE. In 1998–1999, there was a record for isolation of RABV from dogs in Egypt [272]. In 2012–2014, two studies reported the detection of the virus in the brain samples of water buffaloes (*Bubalus bubalis*) exhibiting fever and/or nervous signs. The infection was acquired mostly after being bitten by a fox. The virus was closely related to other street strains of dogs in Egypt, Israel and Jordan [267,273]. In 2015, RABV was isolated from brain tissue of an adult female dog. The dog developed hypersalivation, paralysis, and hyperesthesia consistent with rabies symptoms. The virus was genetically similar to canine RABV circulating in Egypt [274].

Reports of human infection in Egypt have been described from 1904 to 2000s [266] and 30 to 40 annual deaths due to RABV was reported [269,275]. In 1979, a French woman died after corneal transplantation from an Egyptian donor [276]. In 1988, NAMRU3 isolated two street RABV from humans [268]. Genetically, the Egyptian viruses from humans and animals are closely related to those isolated in Israel and the ME [272]. Lastly but not least, the huge number of stray dogs roaming freely with livestock and insufficient vaccination coverage of pets are among the most common hallmarks of the endemic status of RABV in Egypt. 

### 4.3. Rift Valley Fever

Rift Valley fever (RVF) is a vector-borne zoonotic viral disease firstly reported among livestock in Rift Valley of Kenya in the early 1900s. The disease is caused by a RVF virus (RVFV), genus *Phlebovirus*, a member of the RNA family *Bunyaviridae* and was first isolated in 1931. The virus infects mosquitoes, which act as a reservoir, while animals and humans are considered amplifying hosts. RVFV infects a wide spectrum of mammals, causing abortion and mortality. In humans, symptoms range from mild fever, muscle pains and headaches to hepatic failure and death. Direct contact to infected animal blood, aerosol, drinking unpasteurized contaminated milk, or the bite of infected mosquitoes are the major sources for human infection. Vaccination of susceptible animals, restriction of movement (e.g., importation) and reduction or control of mosquitoes’ population are the regular control measures. The disease is common in Africa and the ME [277,278]. 

In the last 40 years, Egypt reported five large outbreaks: in 1977–1978, 1993–1994, 1997, 2000 and 2003 and the largest epizootic was in 1977–1978 [279,280,281,282,283,284]. The primary introduction of RVFV into livestock in these outbreaks in Egypt was mostly linked to importation of animals mainly camels from Africa [280,282,284,285,286,287]. The virus was isolated from various species of domestic animals (e.g., sheep, cows, buffaloes, camels, goats, horses, and rats) as well as humans [288,289].The epizootics of RVF in Egypt were reported every year round. The effects of rainfall and river discharge in addition to optimal constellation of interconnected hydrologic, entomologic (high mosquitoes’ populations), and social conditions were incriminated in the spread of the virus [286,290,291,292,293,294,295]. Moreover, anti-RVFV antibodies were detected in pigs in 2008 [296], domestic and imported cattle and buffaloes in 2009 [87] and non-immunized dairy cattle from different localities in Egypt in 2013–2015 [297]. The reoccurrence of RVF outbreaks from time to time in animals in Egypt challenges the importation control check points and the efficacy of the applied vaccination program [280,281].

In humans, in 1977, RVFV in Egypt caused huge outbreaks including 200,000 human infections and 600 deaths [282,298,299,300,301,302,303,304]. The virus transmitted to eight Swedish United Nations Emergency Forces soldiers serving in Egypt and the Sinai Peninsula [305]. Possible human-to-human transmission was described [302], however the infection mostly occurred by handling infected meat and inhaling natural virus aerosols [287,306]. In 1993, in southern Egypt, 600–1500 human infections associated with ocular disease, fever and headache were reported [279,307]. Serological surveillance indicated that workers at abattoir and sewage treatment plants in several governorates of Egypt possessed RVFV antibodies without showing clinical signs [308,309]. Moreover, in 2008, RVFV antibodies were detected in ~14% of veterinarians and their assistants, butchers and abattoir workers in pigs’ abattoir [296]. 

### 4.4. MERS

Middle East Respiratory Syndrome (MERS) caused by a newly emerging coronavirus (CoV), designated lineage C of Betacoronavirus, in the RNA family *Coronaviridae*. It was firstly reported in Saudi Arabia in a patient with respiratory illness in 2012 [310] and transmitted to several countries not only in the Middle East (ME) but also in Africa, Asia and Europe. The virus is usually transmitted from dromedary camels via direct contact or consumption of milk or medicinal use of camel urine [311]. Also, bats and alpacas were considered reservoir hosts [311,312]. However, limited human-to-human infections have been also reported. The infection can be mild or fatal in those patients with immune system disorders or chronic diseases [313]. 

In Egypt, out of 110 swabs and 52 serums collected in June–December 2013 from clinically healthy imported or locally reared camels in abattoirs, 4 and 48 positive samples were detected, respectively. Attempts to isolate the virus in cell culture were not successful. None of 179 samples collected from workers in these abattoirs were positive by RT-PCR [314]. In another study, in June 2013, all serum samples collected from humans, cows, water buffaloes, goats and sheep were negative for MERS-CoV antibodies. Conversely, 94% of serum samples collected from dromedary camels were positive for MERS-CoV [315]. From June 2014 to February 2016, 2541 sera, 2825 nasal swabs, 114 rectal swabs, 187 milk samples, and 26 urine samples were collected from camels in different sectors in Egypt (importation quarantines, markets, abattoirs, free-roaming herds and farmed breeding herds). Results revealed 71% seropositivity and 15% of other samples were positive by RT-PCR. Seroprevalence was 90% in imported camels and 61% in locally raised camels, likewise RNA detection rates were 21% and 12%, respectively. Both juveniles and adult camels were positive by 82% and 37% seropositivity and similar RT-PCR detection rates of 15% and 16%, respectively [316]. 

In humans, from 2012 to 2015, none of nasopharyngeal and oropharyngeal swabs collected from 3364 returning Egyptian pilgrims were positive for MERS-CoV [317]. To January 2017, only one human case was reported from Egypt in April 2014 [318]. 

### 4.5. Crimean-Congo Hemorrhagic Fever (CCHF)

Crimean-Congo hemorrhagic fever (CCHFV) is a tick-borne virus from the *Bunyaviridae* family. The virus causes severe hemorrhagic fever outbreaks, with a case fatality rate of up to 40%. It is endemic in some African, Middle Eastern and Asian countries. Farm animals like sheep, goats, cattle, camels and ostriches can be infected without showing any clinical signs. Ticks of the genus *Hyalomma* are the principal vector. Humans and animals become infected after being bitten by ticks. Also, animal to human transmission through contact with infected animal blood or tissues, particularly at the abattoirs are common. Therefore, veterinarians, agricultural workers and slaughterhouse workers are most affected [319].

In Egypt in 1976, the antibodies to CCHFV detected in 8.8% camels sera and in 23.1% sheep sera but no antibodies were detected in sera from equines (donkeys, horses and mules), pigs, cows, and buffaloes [320]. In 1986–1987, 14% of serum samples collected from imported camels from Sudan and Kenya into Egypt was positive for CCHFV antibodies. Native livestock including sheep and cows were negative [321]. Between September 2004 and August 2005, 3.83% of cattle, 0.38% of water buffalo, 6.3% of sheep and 1.14% of goats were positive for CCHFV antibodies [322]. In July 2009, ectoparasites removed from freshly slaughtered cattle, buffalo, sheep and camels imported from Sudan and Somalia were negative for CCHFV except five camels were found to harbor ticks carrying RNA from a new CCHFV variant [323]. In 2009 in a serosurvey in domestic and imported livestock, only one cow out of 161 was positive while buffaloes, sheep, and camels were negative for CCHFV antibodies [87]. 

No antibodies were detected in sera from humans in 1976 [320]. The only known human infection with CCHFV in Egypt happened in 1981. An Egyptian virologist died after mouth-pipetting a culture of a CCHFV isolate that he had brought from Iraq [324].

### 4.6. West Nile Fever (WNF) 

West Nile is a remerging ZD caused by West Nile fever virus (WNV), a *Flavivirus* that belongs to the RNA family *Flaviviridae*. The virus was firstly identified in 1937, in Uganda. During the last decade, the incidence of WNV increased worldwide. The virus is transmitted by arthropod vectors and the mosquitoes of the genus *Culex* are main reservoirs [325]. The latter feed on birds especially passerines and thus birds become infected. Migratory birds thought to be responsible for wide spread of WNV and reintroduction from enzootic to new regions [326]. Mosquitoes-birds-mosquitos’ infection is the classical transmission cycle in nature. Accidentally, human infections occur through biting or dealing with contaminated blood or tissues. The infection in birds is mostly subclinical, while humans exhibit mild, if any, illness to fatal West Nile encephalitis particularly in at-risk individuals such as the elderly, immunocompromised and people with chronic illness [327]. Seasonal incidence of WNV outbreaks is linked to higher populations of Culex mosquitoes during summer months in temperate regions and rainy seasons in the tropics. However, infections of humans have been also associated with other factors (e.g., blood transfusion, occupational exposure, etc.) [327].

The Egyptian climate is suitable for the spread of WNV [328] where more than 110 mosquito species and subspecies including Culex species were identified [291,329]. The virus was firstly isolated from mosquitoes in early 1990s [295]. In the period from 1999 to 2002, 15 (0.29%) out of 112,155 examined samples from mosquitoes and sand flies were positive for the WNV. Interestingly, the virus transmitted from mosquitoes to sentinel chickens [330]. It is worth mentioning that more than 150 species of migratory birds visit Egypt annually in addition to 350 resident species of birds [291,329]. Israeli-like WNV was isolated in white storks in Egypt in 1997–2000 suggesting that migrating birds do play a crucial role in geographical spread of the virus [331]. 

The first serological evidence for human infections with WNV in Egypt were reported in 1950 in 22% of children and 61% of adults included in a study conducted by Melnick et al. [332]. In 1968, 14.6% of hospitalized febrile children were linked to WNV infection [333] and 5 out of 133 patients suffering from aseptic meningitis or encephalitis possessed WNV antibodies [334]. In 1969, approximately half of 1113 male University students and 3% of 162 patients in different localities had WNV antibodies [335,336]. Between 1984 and 1985, one out of 55 patients with non-specific fever and myalgia was positive for WNV [337]. In 1989, 3% of school children aged 8–14 years [299] and 45.5% out of 180 examined persons in the Nile Delta were positive for WNV antibodies [338]. WNV had the highest prevalence (54.14%) among other viruses in a serosurvey targeting workers in sewage treatment plants from January to October 1999 [309]. In early 2000s, a Dutch 44-year-old female was infected during a holiday in Egypt [339]. In 1999–2002, WNV was actively circulating in different areas in Egypt causing febrile illness and up to 24% of human serum samples were positive [330]. Currently, the disease is mostly underestimated and scarce data are available, so far.

## 5. Summary and Conclusions

In this review, some aspects of viral, bacterial and parasitic diseases with zoonotic importance in Egypt were summarized. There is a gap of knowledge about the epidemiology of zoonotic diseases in different localities in Egypt, which hinders accurate assessment of the human health burden. Surveillance activity is high for some viral diseases such as influenza and MERS but is still weak or neglected for others particularly at the human-animal interface. Control of diseases in animals depends on the vaccination (e.g., against RVFV, AIVs), anti-microbials for bacteria and antiprotozoal medication against parasitic infection. While some pathogens/diseases are exotic in Egypt (e.g., MERS-CoV and RVFV acquired by importation of camels or WNV from wild birds), others are endemic (e.g., AIV, Rabies, *Schistosoma*, and *Fasciola*). Transboundary transmission of zoonotic agents from Egypt to Europe, Asia and America occurred via different pathways. RABV was reported in the US via falsified vaccination certificate of dogs [274], in Europe via corneal transplantation from an Egyptian donor [276] and in Asia, probably by dog transfer [272]. Also, RVFV infected Swedish soldiers on duty in Egypt in 1977 and 1978 [305]. Egyptian AIV was reported in poultry in neighboring countries most likely due to smuggling of poultry or migratory birds [340]. Approximately 100,000 Egyptians travel to Saudi Arabia in pilgrimage, every year, which is an important risk factor for possible introduction or spread of infections [341].

There are several suggestions to improve the control of zoonoses in animals and humans in Egypt. Enhancing biosecurity and management in animal farms particularly in poultry sectors may reduce the risk of salmonellosis, campylobacteriosis, listeriosis and influenza viruses. Multidrug-resistance of bacteria in animals, due to the misuse of antibiotics in the veterinary sector, is an increasing problem in Egypt. Therefore, regulations for antibiotic application in animals must be enforced to mitigate the serious public health hazard. Vaccination as an alternative approach for the control of bacterial infections in animals, vaccination of stray dogs against RABV and regular investigation of cats for toxoplasmosis should be considered. Longer quarantine periods or restriction of importation of animals, particularly camels, from endemic countries may be effective to reduce introduction of zoonotic viruses. Control of vectors (e.g., mosquitoes), intermediate hosts (e.g., snails) and animal reservoirs (e.g., stray dogs, cats) should be key components in the intervention strategy of zoonoses in Egypt. Improving, providing and upgrading diagnostic techniques in both veterinary and human medicines are fundamental to early detect and contain zoonotic infections. Last but not least, sustainable awareness, education and training targeting groups at high risk (veterinarians, farmers, abattoir workers, nurses, etc.) are of great importance to reduce the burden of zoonoses among Egyptians. Taken together, there is an urgent need for collaborative surveillance and intervention plans for the control of zoonotic diseases in Egypt.

## References

[1] World Health Organization (WHO) Zoonoses and Veterinary Public Health (VPH). http://www.who.int/zoonoses/vph/en/.

[2] Stephen C., Artsob H., Bowie W.R., Drebot M., Fraser E., Leighton T., Morshed M., Ong C., Patrick D. (2004). Perspectives on emerging zoonotic disease research and capacity building in Canada. Can. J. Infect. Dis. Med. Microbiol..

[3] Jones K.E., Patel N.G., Levy M.A., Storeygard A., Balk D., Gittleman J.L., Daszak P. (2008). Global trends in emerging infectious diseases. Nature.

[4] Woolhouse M.E., Gowtage-Sequeria S. (2005). Host range and emerging and reemerging pathogens. Emerg. Infect. Dis..

[5] Taylor L.H., Latham S.M., Woolhouse M.E. (2001). Risk factors for human disease emergence. Philos. Trans. R. Soc. Lond. B Biol. Sci..

[6] Coulibaly N.D., Yameogo K.R. (2000). Prevalence and control of zoonotic diseases: Collaboration between public health workers and veterinarians in Burkina Faso. Acta Trop..

[7] Metzgar D., Baynes D., Myers C.A., Kammerer P., Unabia M., Faix D.J., Blair P.J. (2010). Initial identification and characterization of an emerging zoonotic influenza virus prior to pandemic spread. J. Clin. Microbiol..

[8] Leslie M.J., McQuiston J.H. (1992). Emerging infections: Microbial threats to health in the United States. Washington. Infectious Disease Surveillance.

[9] Glaser C.A., Angulo F.J., Rooney J.A. (1994). Animal-associated opportunistic infections among persons infected with the human immunodeficiency virus. Clin. Infect. Dis..

[10] Tauxe R.V. (1997). Emerging foodborne diseases: An evolving public health challenge. Emerg. Infect. Dis..

[11] Esch K.J., Petersen C.A. (2013). Transmission and epidemiology of zoonotic protozoal diseases of companion animals. Clin. Microbiol. Rev..

[12] Cantas L., Suer K. (2014). Review: The important bacterial zoonoses in “one health” concept. Front. Public Health.

[13] McDaniel C.J., Cardwell D.M., Moeller R.B., Gray G.C. (2014). Humans and cattle: A review of bovine zoonoses. Vector Borne Zoonotic Dis..

[14] Agunos A., Pierson F.W., Lungu B., Dunn P.A., Tablante N. (2016). Review of nonfoodborne zoonotic and potentially zoonotic poultry diseases. Avian Dis..

[15] Kassem I.I., Kehinde O.O., Helmy Y.A., Kumar A., Chandrashekhar K., Pina-Mimbela R., Rajashekara G., Mei Soon J., Manning L., Wallace C.A. (2016). Campylobacter in poultry: The conundrums of highly adaptable and ubiquitous foodborne pathogens. Foodborne Diseases: Case Studies of Outbreaks in the Agri-Food Industries.

[16] On S.L. (2001). Taxonomy of *Campylobacter*, *Arcobacter*, *Helicobacter* and related bacteria: Current status, future prospects and immediate concerns. Symp. Ser. Soc. Appl. Microbiol..

[17] Silva J., Leite D., Fernandes M., Mena C., Gibbs P.A., Teixeira P. (2011). *Campylobacter* spp. as a Foodborne Pathogen: A Review. Front. Microbiol..

[18] Lin J. (2009). Novel approaches for *Campylobacter* control in poultry. Foodborne Pathog. Dis..

[19] Barakat A., Sobhy M., El Fadaly H.A.A., Rabie N., Khalifa N., Hassan E., Kotb M., Amin Girh Z., Sedeek D., Zaki M. (2015). Zoonotic Hazards of Campylobacteriosis in some areas in Egypt. Life Sci. J..

[20] El Fadaly H., Barakat A., Ahmed S., Abd El-Razik K., Omara S., Ezzat E., Zaki M. (2016). Zoonotic concern of *Campylobacter jejuni* in raw and ready-to-eat barbeque chickens along with Egyptian handlers and consumers via molecular and immunofluorescent characterization. Der Pharma Chemica.

[21] El-Sharoud W.M. (2009). Prevalence and survival of *Campylobacter* in Egyptian dairy products. Food Res. Int..

[22] El-Zamkan M.A., Hameed K.G. (2016). Prevalence of *Campylobacter jejuni* and *Campylobacter coli* in raw milk and some dairy products. Vet. World.

[23] EL-Shahawy H., Sobhy M., Abd El-Gaied S. (2009). Incidence of *Campylobacter* and anaerobic bacteria among apparently healthy and diarrheic camel calves. Egypt. J. Comp. Pathol. Clin. Pathol..

[24] El-Tras W.F., Holt H.R., Tayel A.A., El-Kady N.N. (2015). *Campylobacter* infections in children exposed to infected backyard poultry in Egypt. Epidemiol. Infect..

[25] Khalifa N., Afify J., Rabie N. (2013). Zoonotic and molecular characterizations of *Campylobacter jejuni* and *Campylobacter coli* isolated from beef cattle and children. Glob. Vet..

[26] Omara S., El Fadaly H., Barakat A. (2015). Public health hazard of zoonotic *Campylobacter jejuni* reference to Egyptian regional and seasonal variations. Res. J. Microbiol..

[27] Awadallah M., Ahmed H., El-Gedawy A., Saad A. (2014). Molecular identification of *C. jejuni* and *C. coli* in chicken and humans, at Zagazig, Egypt, with reference to the survival of *C. jejuni* in chicken meat at refrigeration and freezing temperatures. Int. Food Res. J..

[28] Ahmed H.A., El Hofy F.I., Ammar A.M., Abd El Tawab A.A., Hefny A.A. (2015). ERIC-PCR genotyping of some Campylobacter jejuni isolates of chicken and human origin in Egypt. Vector Borne Zoonotic Dis..

[29] Pazzaglia G., Bourgeois A.L., Mourad A.S., Gaafar T., Diab A.S., Hebert A., Churilla A., Murphy J.R. (1995). *Campylobacter* diarrhea in Alexandria, Egypt. J. Egypt. Public Health Assoc..

[30] Mohran Z., Guerry P., Lior H., Murphy J., el-Gendy A., Mikhail M., Oyofo B. (1996). Restriction fragment length polymorphism of flagellin genes of *Campylobacter jejuni* and/or *C. coli* isolates from Egypt. J. Clin. Microbiol..

[31] Gould L.H., Walsh K.A., Vieira A.R., Herman K., Williams I.T., Hall A.J., Cole D., Centers for Disease Control and Prevention (2013). Surveillance for foodborne disease outbreaks-United States, 1998–2008. MMWR Surveill. Summ..

[32] Collard J.M., Bertrand S., Dierick K., Godard C., Wildemauwe C., Vermeersch K., Duculot J., Van Immerseel F., Pasmans F., Imberechts H. (2008). Drastic decrease of *Salmonella* Enteritidis isolated from humans in Belgium in 2005, shift in phage types and influence on foodborne outbreaks. Epidemiol. Infect..

[33] Tarabees R., Elsayed M.S.A., Shawish R., Basiouni S., Shehata A.A. (2017). Isolation and characterization of *Salmonella* Enteritidis and *Salmonella* Typhimurium from chicken meat in Egypt. J. Infect. Dev. Ctries..

[34] Kassem I., Helmy Y.A., Kashoma I.P., Rajashekara G. (2016). The Emergence of Antibiotic Resistance on Poultry Farms.

[35] El-Sharkawy H., Tahoun A., El-Gohary A.E.A., El-Abasy M., El-Khayat F., Gillespie T., Kitade Y., Hafez H.M., Neubauer H., El-Adawy H. (2017). Epidemiological, molecular characterization and antibiotic resistance of *Salmonella enterica* serovars isolated from chicken farms in Egypt. Gut Pathog..

[36] Ammar A., Mohamed A., Abd El-Hamid M., El-Azzouny M. (2016). Virulence genotypes of clinical *Salmonella* Serovars from broilers in Egypt. J. Infect. Dev. Ctries..

[37] Abdel-Aziz N. (2016). Detection of *Salmonella* species in chicken carcasses using genus specific primer belong to *inv*A gene in Sohag city, Egypt. Vet. World.

[38] El-Tras W.F., Tayel A.A., Samir A. (2010). Potential zoonotic pathways of *Salmonella enteritidis* in laying farms. Vector Borne Zoonotic Dis..

[39] Osman K.M., Mehrez M., Erfan A.M., Nayerah A. (2013). *Salmonella enterica* isolated from pigeon (*Columba livia*) in Egypt. Foodborne Pathog. Dis..

[40] Younis E.E., Ahmed A.M., El-Khodery S.A., Osman S.A., El-Naker Y.F. (2009). Molecular screening and risk factors of enterotoxigenic *Escherichia coli* and *Salmonella* spp. in diarrheic neonatal calves in Egypt. Res. Vet. Sci..

[41] Osman K.M., Marouf S.H., Zolnikov T.R., AlAtfeehy N. (2014). Isolation and characterization of *Salmonella enterica* in day-old ducklings in Egypt. Pathog. Glob. Health.

[42] Ghoneim N.H., Abdel-Moein K.A., Zaher H. (2017). Camel as a transboundary vector for emerging exotic *Salmonella* serovars. Pathog. Glob. Health.

[43] Ahmed A.M., Shimamoto T., Shimamoto T. (2014). Characterization of integrons and resistance genes in multidrug-resistant *Salmonella enterica* isolated from meat and dairy products in Egypt. Int. J. Food Microbiol..

[44] Ahmed A.M., Shimamoto T. (2014). Isolation and molecular characterization of *Salmonella enterica*, *Escherichia coli* O157:H7 and *Shigella* spp. from meat and dairy products in Egypt. Int. J. Food Microbiol..

[45] Gwida M., Al-Ashmawy M. (2014). Culture versus PCR for *Salmonella* species identification in some dairy products and dairy handlers with special concern to its zoonotic importance. Vet. Med. Int..

[46] Bakr W., El Sayed A., El Shamy H., Amine A. (2013). Is it safe to eat raw seafood? Prevalence of *Salmonella* in some seafood products sold in Alexandria markets. J. Egypt. Public Health Assoc..

[47] Saleh F.O.I., Ahmed H.A., Khairy R.M.M., Abdelwahab S.F. (2014). Increased quinolone resistance among typhoid *Salmonella* isolated from Egyptian patients. J. Infect. Dev. Ctries..

[48] Osman K.M., Marouf S.H., Alatfeehy N. (2013). Antimicrobial resistance and virulence-associated genes of *Salmonella enterica* subsp. enterica serotypes Muenster, Florian, Omuna, and Noya strains isolated from clinically diarrheic humans in Egypt. Microb. Drug Resist..

[49] Ahmed H.A., El-Hofy F.I., Shafik S.M., Abdelrahman M.A., Elsaid G.A. (2016). Characterization of virulence-associated genes, antimicrobial resistance genes, and class 1 integrons in salmonella enterica serovar Typhimurium isolates from chicken meat and humans in Egypt. Foodborne Pathog. Dis..

[50] Pappas G., Papadimitriou P., Akritidis N., Christou L., Tsianos E.V. (2006). The new global map of human brucellosis. Lancet Infect. Dis..

[51] Corbel M.J. (1997). Brucellosis: An overview. Emerg. Infect. Dis..

[52] Garin-Bastuji B., Blasco J.M., Grayon M., Verger J.M. (1998). *Brucella melitensis* infection in sheep: Present and future. Vet. Res..

[53] Refai M. (2002). Incidence and control of brucellosis in the Near East region. Vet. Microbiol..

[54] Eltholth M.M., Hegazy Y.M., El-Tras W.F., Bruce M., Rushton J. (2016). Temporal analysis and costs of ruminant brucellosis control programme in Egypt between 1999 and 2011. Transbound. Emerg. Dis..

[55] Elmonir W., Hegazy Y., AbdelHamid N., Elbauomy E. (2016). Brucellosis at the human-animal Interface in Kafrelsheikh Governorate, Egypt. Alex. J. Vet. Sci..

[56] El-Hady A., Ahmed M., Saleh M., Younis E. (2016). Seroprevalence and molecular epidemiology of brucellosis in cattle in Egypt. Adv. Dairy Res..

[57] Kaoud H., Zaki M., El-Dahshan A., Nasr S. (2010). Epidemiology of Brucellosis among farm animals. Nat. Sci..

[58] Jennings G.J., Hajjeh R.A., Girgis F.Y., Fadeel M.A., Maksoud M.A., Wasfy M.O., El-Sayed N., Srikantiah P., Luby S.P., Earhart K. (2007). Brucellosis as a cause of acute febrile illness in Egypt. Trans. R. Soc. Trop. Med. Hyg..

[59] Garcia A., Fox J.G., Besser T.E. (2010). Zoonotic enterohemorrhagic *Escherichia coli*: A One Health perspective. ILAR J..

[60] Sallam K.I., Mohammed M.A., Ahdy A.M., Tamura T. (2013). Prevalence, genetic characterization and virulence genes of sorbitol-fermenting *Escherichia coli* O157:H- and *E. coli* O157:H7 isolated from retail beef. Int. J. Food Microbiol..

[61] Ombarak R.A., Hinenoya A., Awasthi S.P., Iguchi A., Shima A., Elbagory A.R., Yamasaki S. (2016). Prevalence and pathogenic potential of *Escherichia coli* isolates from raw milk and raw milk cheese in Egypt. Int. J. Food Microbiol..

[62] Hussein A.H., Ghanem I.A., Eid A.A., Ali M.A., Sherwood J.S., Li G., Nolan L.K., Logue C.M. (2013). Molecular and phenotypic characterization of *Escherichia coli* isolated from broiler chicken flocks in Egypt. Avian Dis..

[63] Ramadan H., Awad A., Ateya A. (2016). Detection of phenotypes, virulence genes and phylotypes of avian pathogenic and human diarrheagenic *Escherichia coli* in Egypt. J. Infect. Dev. Ctries..

[64] Ahmed S.F., Shaheen H.I., Abdel-Messih I.A., Mostafa M., Putnam S.D., Kamal K.A., Sayed A.N., Frenck R.W., Sanders J.W., Klena J.D. (2014). The epidemiological and clinical characteristics of diarrhea associated with enteropathogenic, enteroaggregative and diffuse-adherent *Escherichia coli* in Egyptian children. J. Trop. Pediatr..

[65] Elsherif R.H., Ismail D.K., El-Kholy Y.S., Gohar N.M., Elnagdy S.M., Elkraly O.A. (2016). Integron-mediated multidrug resistance in extended-spectrum beta-lactamase-producing *Escherichia coli* and *Klebsiella pneumoniae* isolated from fecal specimens in Egypt. J. Egypt. Public Health Assoc..

[66] Braun S.D., Ahmed M.F., El-Adawy H., Hotzel H., Engelmann I., Weiss D., Monecke S., Ehricht R. (2016). Surveillance of extended-spectrum beta-lactamase-producing Escherichia coli in dairy cattle farms in the Nile Delta, Egypt. Front. Microbiol..

[67] Ramaswamy V., Cresence V.M., Rejitha J.S., Lekshmi M.U., Dharsana K.S., Prasad S.P., Vijila H.M. (2007). Listeria—Review of epidemiology and pathogenesis. J. Microbiol. Immunol. Infect..

[68] El-Taweel G.E., Shaban A.M. (2001). Microbiological quality of drinking water at eight water treatment plants. Int. J. Environ. Health Res..

[69] El-Shenawy M.A., El-Shenawy M.A. (2006). *Listeria* spp. in the coastal environment of the Aqaba Gulf, Suez Gulf and the Red Sea. Epidemiol. Infect..

[70] El-Shenawy M., El-Shenawy M., Manes J., Soriano J.M. (2011). *Listeria* spp. in Street-Vended Ready-to-Eat Foods. Interdiscip. Perspect. Infect. Dis..

[71] Ahmed H.A., Hussein M.A., El-Ashram A.M.M. (2013). Seafood a potential source of some zoonotic bacteria in Zagazig, Egypt, with the molecular detection of *Listeria monocytogenes* virulence genes. Vet. Ital..

[72] Osman K.M., Samir A., Orabi A., Zolnikov T.R. (2014). Confirmed low prevalence of *Listeria* mastitis in she-camel milk delivers a safe, alternative milk for human consumption. Acta Trop..

[73] Osman K.M., Zolnikov T.R., Samir A., Orabi A. (2014). Prevalence, pathogenic capability, virulence genes, biofilm formation, and antibiotic resistance of *Listeria* in goat and sheep milk confirms need of hygienic milking conditions. Pathog. Glob. Health.

[74] Osman K.M., Samir A., Abo-Shama U.H., Mohamed E.H., Orabi A., Zolnikov T. (2016). Determination of virulence and antibiotic resistance pattern of biofilm producing *Listeria* species isolated from retail raw milk. BMC Microbiol..

[75] El-Shamy H.A., El-Molla A.H., Abou Donia S.A., Medhagi A.K. (1993). Detection and survival of *Listeria monocytogenes* in milk and dairy products. J. Egypt. Public Health Assoc..

[76] Dahshan H., Merwad A.M., Mohamed T.S. (2016). Listeria species in broiler poultry farms: Potential public health hazards. J. Microbiol. Biotechnol..

[77] Reda W.W., Abdel-Moein K., Hegazi A., Mohamed Y., Abdel-Razik K. (2016). *Listeria monocytogenes*: An emerging food-borne pathogen and its public health implications. J. Infect. Dev. Ctries..

[78] El Sayed Zaki M., El Shabrawy W.O., El-Eshmawy M.M., Aly Eletreby S. (2011). The high prevalence of *Listeria monocytogenes* peritonitis in cirrhotic patients of an Egyptian Medical Center. J. Infect. Public Health.

[79] Abd El-Malek A.M., Ali S.F.H., Hassanein R., Moemen A.M., Elsayh K.I. (2010). Occurrence of Listeria species in meat, chicken products and human stools in Assiut city, Egypt with PCR use for rapid identification of Listeria monocytogenes. Vet. World.

[80] Vanderburg S., Rubach M.P., Halliday J.E., Cleaveland S., Reddy E.A., Crump J.A. (2014). Epidemiology of *Coxiella burnetii* infection in Africa: A OneHealth systematic review. PLoS Negl. Trop. Dis..

[81] Maurin M., Raoult D. (1999). Q fever. Clin. Microbiol. Rev..

[82] Ghoneim N., Abdel-Moein K. (2012). Seroprevalence of *Coxiella burnetii* antibodies among farm animals and human contacts in Egypt. J. Am. Sci..

[83] Sixl W., Sebek Z., Kock M., Marth E., Withalm H. (1989). Serologic studies of domestic animals for listeriosis, Q-fever, and brucellosis in Cairo. Geogr. Med. Suppl..

[84] Gwida M., El-Ashker M., El-Diasty M., Engelhardt C., Khan I., Neubauer H. (2014). Q fever in cattle in some Egyptian Governorates: A preliminary study. BMC Res. Notes.

[85] El-Mahallawy H., Abou-Eisha A., Fadel H. (2012). *Coxiella burnetii* infections among small ruminants in Ismailia Governorate. SCVMJ.

[86] Mazyad S., Hafez A. (2007). Q fever (*Coxiella burnetii*) among man and farm animals in North Sinai, Egypt. J. Egypt. Soc. Parasitol..

[87] Horton K.C., Wasfy M., Samaha H., Abdel-Rahman B., Safwat S., Abdel Fadeel M., Mohareb E., Dueger E. (2014). Serosurvey for zoonotic viral and bacterial pathogens among slaughtered livestock in Egypt. Vector Borne Zoonotic Dis..

[88] Botros B., Soliman A., Salib A., Olson J., Hibbs R., Williams J., Darwish M., el Tigani A., Watts D. (1995). *Coxiella burnetii* antibody prevalences among human populations in north-east Africa determined by enzyme immunoassay. J. Trop. Med. Hyg..

[89] Abdel-Moein K.A., Hamza D.A. (2017). The burden of *Coxiella burnetii* among aborted dairy animals in Egypt and its public health implications. Acta Trop..

[90] Chitsulo L., Engels D., Montresor A., Savioli L. (2000). The global status of schistosomiasis and its control. Acta Trop..

[91] World Health Organization (WHO) Schistosomiasis. http://www.who.int/zoonoses/vph/en/.

[92] Steinmann P., Keiser J., Bos R., Tanner M., Utzinger J. (2006). Schistosomiasis and water resources development: Systematic review, meta-analysis, and estimates of people at risk. Lancet Infect. Dis..

[93] CDC Schistosomiasis. https://www.cdc.gov/parasites/schistosomiasis/.

[94] Colley D.G., Bustinduy A.L., Secor W.E., King C.H. (2014). Human schistosomiasis. Lancet.

[95] Zoni A.C., Catala L., Ault S.K. (2016). Schistosomiasis Prevalence and Intensity of Infection in Latin America and the Caribbean Countries, 1942–2014: A Systematic Review in the Context of a Regional Elimination Goal. PLoS Negl. Trop. Dis..

[96] Gryseels B., Polman K., Clerinx J., Kestens L. (2006). Human schistosomiasis. Lancet.

[97] Van der Werf M.J., de Vlas S.J., Brooker S., Looman C.W., Nagelkerke N.J., Habbema J.D., Engels D. (2003). Quantification of clinical morbidity associated with schistosome infection in sub-Saharan Africa. Acta Trop..

[98] Deelder A.M., Miller R.L., de Jonge N., Krijger F.W. (1990). Detection of schistosome antigen in mummies. Lancet.

[99] Ziskind B. (2009). Urinary schistosomiasis in ancient Egypt. Nephrol. Ther..

[100] Cox F.E. (2002). History of human parasitology. Clin. Microbiol. Rev..

[101] Bustinduy A.L., Parraga I.M., Thomas C.L., Mungai P.L., Mutuku F., Muchiri E.M., Kitron U., King C.H. (2013). Impact of polyparasitic infections on anemia and undernutrition among Kenyan children living in a Schistosoma haematobium-endemic area. Am. J. Trop. Med. Hyg..

[102] Barakat R.M. (2013). Epidemiology of Schistosomiasis in Egypt: Travel through time: Review. J. Adv. Res..

[103] Bakr I.M., Arafa N.A., Ahmed M.A., Mostafa Mel H., Mohamed M.K. (2009). Prevalence of intestinal parasitosis in a rural population in Egypt, and its relation to socio-demographic characteristics. J. Egypt. Soc. Parasitol..

[104] Talaat M., El-Ayyat A., Sayed H.A., Miller F.D. (1999). Emergence of Schistosoma mansoni infection in upper Egypt: The Giza governorate. Am J. Trop. Med. Hyg..

[105] Scott J. (1937). The incidence and distribution of the human schistosomiasis in Egypt. Am. J. Hyg..

[106] Miller F., Hussein M., Mancy K.H., Hilbert M.S., Monto A.S., Barakat R.M. (1981). An epidemiological study of *Schistosoma haematobium* and *S. mansoni* infection in thirty-five rural Egyptian villages. Trop. Geogr. Med..

[107] Michelson M.K., Azziz F.A., Gamil F.M., Wahid A.A., Richards F.O., Juranek D.D., Habib M.A., Spencer H.C. (1993). Recent trends in the prevalence and distribution of schistosomiasis in the Nile delta region. Am. J. Trop. Med. Hyg..

[108] Cline B.L., Richards F.O., el Alamy M.A., el Hak S., Ruiz-Tiben E., Hughes J.M., McNeeley D.F. (1989). 1983 Nile Delta schistosomiasis survey: 48 years after Scott. Am. J. Trop. Med. Hyg..

[109] El-Khoby T., Galal N., Fenwick A., Barakat R., El-Hawey A., Nooman Z., Habib M., Abdel-Wahab F., Gabr N.S., Hammam H.M. (2000). The epidemiology of schistosomiasis in Egypt: Summary findings in nine governorates. Am. J. Trop. Med. Hyg..

[110] Farooq M., Nielsen J., Samaan S.A., Mallah M.B., Allam A.A. (1966). The epidemiology of Schistosoma haematobium and S. mansoni infections in the Egypt-49 project area. 2. Prevalence of bilharziasis in relation to personal attributes and habits. Bull. World Health Organ..

[111] Abdel-Wahab M.F., Esmat G., Medhat E., Narooz S., Ramzy I., El-Boraey Y., Strickland G.T. (2000). The epidemiology of schistosomiasis in Egypt: Menofia Governorate. Am. J. Trop. Med. Hyg..

[112] Abdel-Wahab M.F., Esmat G., Ramzy I., Narooz S., Medhat E., Ibrahim M., El-Boraey Y., Strickland G.T. (2000). The epidemiology of schistosomiasis in Egypt: Fayoum Governorate. Am. J. Trop. Med. Hyg..

[113] Habib M., Abdel Aziz F., Gamil F., Cline B.L. (2000). The epidemiology of schistosomiasis in Egypt: Qalyubia Governorate. Am. J. Trop. Med. Hyg..

[114] Nooman Z.M., Hasan A.H., Waheeb Y., Mishriky A.M., Ragheb M., Abu-Saif A.N., Abaza S.M., Serwah A.A., El-Gohary A., Saad A. (2000). The epidemiology of schistosomiasis in Egypt: Ismailia governorate. Am. J. Trop. Med. Hyg..

[115] Hammam H.M., Allam F.A., Moftah F.M., Abdel-Aty M.A., Hany A.H., Abd-El-Motagaly K.F., Nafeh M.A., Khalifa R., Mikhail N.N., Talaat M. (2000). The epidemiology of schistosomiasis in Egypt: Assiut governorate. Am. J. Trop. Med. Hyg..

[116] Hammam H.M., Zarzour A.H., Moftah F.M., Abdel-Aty M.A., Hany A.H., El-Kady A.Y., Nasr A.M., Abd-El-Samie A., Qayed M.H., Mikhail N.N. (2000). The epidemiology of schistosomiasis in Egypt: Qena governorate. Am. J. Trop. Med. Hyg..

[117] El-Hawey A.M., Amr M.M., Abdel-Rahman A.H., El-Ibiary S.A., Agina A.M., Abdel-Hafez M.A., Waheeb A.A., Hussein M.H., Strickland G.T. (2000). The epidemiology of schistosomiasis in Egypt: Gharbia Governorate. Am. J. Trop. Med. Hyg..

[118] Medhat A., Abdel-Aty M.A., Nafeh M., Hammam H., Abdel-Samia A., Strickland G.T. (1993). Foci of Schistosoma mansoni in Assiut province in middle Egypt. Trans. R. Soc. Trop. Med. Hyg..

[119] Abdel-Wahab M.F., Yosery A., Narooz S., Esmat G., el Hak S., Nasif S., Strickland G.T. (1993). Is Schistosoma mansoni replacing Schistosoma haematobium in the Fayoum?. Am. J. Trop. Med. Hyg..

[120] El Khoby T., Galal N., Fenwick A. (1998). The USAID/Government of Egypt’s Schistosomiasis Research Project (SRP). Parasitol. Today.

[121] Barakat A., Halawa E.F. (2013). Household costs of seeking outpatient care in Egyptian children with diarrhea: A cross-sectional study. Pan Afr. Med. J..

[122] Haggag A.A., Rabiee A., Abd Elaziz K.M., Gabrielli A.F., Abdel Hay R., Ramzy R.M. (2017). Mapping of Schistosoma mansoni in the Nile Delta, Egypt: Assessment of the prevalence by the circulating cathodic antigen urine assay. Acta Trop..

[123] Ross A.G., Chau T.N., Inobaya M.T., Olveda R.M., Li Y., Harn D.A. (2017). A new global strategy for the elimination of schistosomiasis. Int. J. Infect. Dis..

[124] Elmorshedy H., Bergquist R., El-Ela N.E., Eassa S.M., Elsakka E.E., Barakat R. (2015). Can human schistosomiasis mansoni control be sustained in high-risk transmission foci in Egypt?. Parasit Vectors.

[125] World Health Organization (WHO) (2007). Report of the WHO Informal Meeting on Use of Triclabendazole in Fascioliasis Control.

[126] Haridy F.M., Morsy T.A., Gawish N.I., Antonios T.N., Abdel Gawad A.G. (2002). The potential reservoir role of donkeys and horses in zoonotic fascioliasis in Gharbia Governorate, Egypt. J. Egypt. Soc. Parasitol..

[127] Mas-Coma S., Valero M.A., Bargues M.D. (2014). Fascioliasis. Adv. Exp. Med. Biol..

[128] Mas-Coma S., Valero M.A., Bargues M.D. (2009). Chapter 2. Fasciola, lymnaeids and human fascioliasis, with a global overview on disease transmission, epidemiology, evolutionary genetics, molecular epidemiology and control. Adv. Parasitol..

[129] World Health Organization (WHO) Foodborne Trematodiases. http://www.who.int/mediacentre/factsheets/fs368/en/.

[130] Esteban J.G., Gonzalez C., Curtale F., Munoz-Antoli C., Valero M.A., Bargues M.D., el-Sayed M., el-Wakeel A.A., Abdel-Wahab Y., Montresor A. (2003). Hyperendemic fascioliasis associated with schistosomiasis in villages in the Nile Delta of Egypt. Am. J. Trop. Med. Hyg..

[131] Dietrich C.F., Kabaalioglu A., Brunetti E., Richter J. (2015). Fasciolosis. Z. Gastroenterol..

[132] Haseeb A.N., el-Shazly A.M., Arafa M.A., Morsy A.T. (2002). A review on fascioliasis in Egypt. J. Egypt. Soc. Parasitol..

[133] Lotfy W.M. (2014). Climate change and epidemiology of human parasitosis in Egypt: A review. J. Adv. Res..

[134] Spithill T.W., Smooker P.M., Copeman D.B., Dalton J.P. (1999). Fasciola gigantica: Epidemiology, control, immunology and molecular biology. Fasciolosis.

[135] Soliman M.S., Angelico M., Rocchi G. (1998). Control of veterinary fascioliasis. Infectious Diseases and Public Health. A Research and Clinical Update.

[136] Mas-Coma S., Bargues M.D. (1997). Human liver flukes: A review. Res. Rev. Parasitol..

[137] Farag H.F. (1998). Human fascioliasis in some countries of the Eastern Mediterranean Region. East Mediterr. Health J..

[138] Haridy F.M., El-Sherbiny G.T., Morsy T.A. (2006). Some parasitic flukes infecting farm animals in Al-Santa Center, Gharbia Governorate, Egypt. J. Egypt. Soc. Parasitol..

[139] Soliman M.F. (2008). Epidemiological review of human and animal fascioliasis in Egypt. J. Infect. Dev. Ctries..

[140] Haridy F.M., Ibrahim B.B., Morsy T.A., el-Sharkawy I.M. (1999). Fascioliasis an increasing zoonotic disease in Egypt. J. Egypt. Soc. Parasitol..

[141] El-Shazly A.M., El-Wafa S.A., Haridy F.M., Soliman M., Rifaat M.M., Morsy T.A. (2002). Fascioliasis among live and slaugthered animals in nine centers of Dakahlia Governorate. J. Egypt. Soc. Parasitol..

[142] Hussein A.A., Khalifa R.M.A. (2010). Fascioliasis prevalences among animals and human in Upper Egypt. J. King Saud Univ. Sci..

[143] Amer S., ElKhatam A., Zidan S., Feng Y., Xiao L. (2016). Identity of Fasciola spp. in sheep in Egypt. Parasit. Vectors.

[144] Morsy T.A., Salem H.S., Haridy F.M., Rifaat M.M., Abo-Zenadah N.Y., Adel el-Kadi M. (2005). Farm animals’ fascioliasis in Ezbet El-Bakly (Tamyia Center) Al-Fayoum Governorate. J. Egypt. Soc. Parasitol..

[145] Abouzeid N.Z., Selim A.M., El-Hady K.M. (2010). Prevalence of gastrointestinal parasites infections in sheep in the Zoo garden and Sinai district and study the efficacy of anthelmintic drugs in the treatment of these parasites. J. Am. Sci..

[146] El-Shazly A.M., Abdel-Magied A.A., El-Nahas H.A., El-Metwaly M.S., Morsy T.A., El Sharkawy E.M., Morsy A.T. (2005). On the main reservoir host of Fasciola in Dakahlia Governorate, Egypt. J. Egypt. Soc. Parasitol..

[147] World Health Organization (WHO) (1995). Control of Foodborne Trematode Infections. WHO Technical Report Series.

[148] El Shazly A.M., Awad S.E., Sultan D.M., Sadek G.S., Khalil H.H., Morsy T.A. (2006). Intestinal parasites in Dakahlia governorate, with different techniques in diagnosing protozoa. J. Egypt. Soc. Parasitol..

[149] Curtale F., Abd-El Wahab Hassanein Y., El Wakeel A., Barduagni P., Savioli L. (2003). The school health programme in Behera: An integrated helminth control programme at Governorate level in Egypt. Acta Trop..

[150] Farag H.F., Barakat R.M., Ragab M., Omar E. (1979). A focus of human fascioliasis in the Nile Delta, Egypt. J. Trop. Med. Hyg..

[151] Hassan M.M., Moustafa N.E., Mahmoud L.A., Abbaza B.E., Hegab M.H. (1995). Prevalence of Fasciola infection among school children in Sharkia Governorate, Egypt. J. Egypt. Soc. Parasitol..

[152] Fawzi M., El-Sahn A.A., Ibrahim H.F., Shehata A.I. (2004). Vegetable-transmitted parasites among inhabitants of El-Prince, Alexandria and its relation to housewives’ knowledge and practices. J. Egypt. Public Health Assoc..

[153] Salem A.I., Osman M.M., el-Daly S., Farahat A. (1993). Studies on Lymnaea snails and their trematode parasites in Abis II village, Alexandria. J. Egypt. Soc. Parasitol..

[154] Curtale F., Hassanein Y.A., Savioli L. (2005). Control of human fascioliasis by selective chemotherapy: Design, cost and effect of the first public health, school-based intervention implemented in endemic areas of the Nile Delta, Egypt. Trans. R. Soc. Trop. Med. Hyg..

[155] El-Bahy M.M., Mahgoub A.M., Taher E.E. (2014). Contributions on human fascioliasis and its snail intermediate host in Nile Delta, Egypt. Int. J. Basic Appl. Sci..

[156] Mekky M.A., Tolba M., Abdel-Malek M.O., Abbas W.A., Zidan M. (2015). Human fascioliasis: A re-emerging disease in upper Egypt. Am. J. Trop. Med. Hyg..

[157] Dillingham R.A., Lima A.A., Guerrant R.L. (2002). Cryptosporidiosis: Epidemiology and impact. Microbes Infect..

[158] Tyzzer E.E. (1910). An extracellular Coccidium, Cryptosporidium Muris (Gen. Et Sp. Nov.), of the gastric Glands of the Common Mouse. J. Med. Res..

[159] Meisel J.L., Perera D.R., Meligro C., Rubin C.E. (1976). Overwhelming watery diarrhea associated with a cryptosporidium in an immunosuppressed patient. Gastroenterology.

[160] Meuten D.J., Van Kruiningen H.J., Lein D.H. (1974). Cryptosporidiosis in a calf. J. Am. Vet. Med. Assoc..

[161] Heine J., Pohlenz J.F., Moon H.W., Woode G.N. (1984). Enteric lesions and diarrhea in gnotobiotic calves monoinfected with Cryptosporidium species. J. Infect. Dis..

[162] MacKenzie W.R., Hoxie N.J., Proctor M.E., Gradus M.S., Blair K.A., Peterson D.E. (1994). A massive outbreak in Milwaukee of Cryptosporidium infection transmitted through the public water supply. N. Engl. J. Med..

[163] Peng M.M., Xiao L., Freeman A.R., Arrowood M.J., Escalante A.A., Weltman A.C., Ong C.S., Mac Kenzie W.R., Lal A.A., Beard C.B. (1997). Genetic polymorphism among Cryptosporidium parvum isolates: Evidence of two distinct human transmission cycles. Emerg. Infect. Dis..

[164] Thompson R.C., Palmer C.S., O’Handley R. (2008). The public health and clinical significance of Giardia and Cryptosporidium in domestic animals. Vet. J..

[165] Xiao L., Ryan U.M. (2004). Cryptosporidiosis: An update in molecular epidemiology. Curr. Opin. Infect. Dis..

[166] Chen X.M., Keithly J.S., Paya C.V., LaRusso N.F. (2002). Cryptosporidiosis. N. Engl. J. Med..

[167] Chen W., Harp J.A., Harmsen A.G. (2003). Cryptosporidium parvum infection in gene-targeted B cell-deficient mice. J. Parasitol..

[168] Helmy Y.A., Krucken J., Nockler K., von Samson-Himmelstjerna G., Zessin K.H. (2014). Comparison between two commercially available serological tests and polymerase chain reaction in the diagnosis of Cryptosporidium in animals and diarrhoeic children. Parasitol. Res..

[169] Abou-Eisha A.M., Hussein M.M., Abdel-Aal A.A., Saleh R.E. (2000). Cryptosporidium in drinking water sources and its zoonotic importance. Minufiya Vet. J..

[170] Shoukry N.M., Dawoud H.A., Haridy F.M. (2009). Studies on zoonotic cryptosporidiosis parvum in Ismailia Governorate, Egypt. J. Egypt. Soc. Parasitol..

[171] Abou-Eisha A.M. (1994). Cryptosporidial infection in man and farm animals in Ismailia governorate. Vet. Med. J. Giza.

[172] Hassanein S.M., Abd-El-Latif M.M., Hassanin O.M., Abd-El-Latif L.M., Ramadan N.I. (2012). Cryptosporidium gastroenteritis in Egyptian children with acute lymphoblastic leukemia: Magnitude of the problem. Infection.

[173] Amer S., Honma H., Ikarashi M., Tada C., Fukuda Y., Suyama Y., Nakai Y. (2010). Cryptosporidium genotypes and subtypes in dairy calves in Egypt. Vet. Parasitol..

[174] El-Khodery S.A., Osman S.A. (2008). Cryptosporidiosis in buffalo calves (*Bubalus bubalis*): Prevalence and potential risk factors. Trop. Anim. Health Prod..

[175] Abd-El-Wahed M.M. (1999). Cryptosporidium infection among sheep in Qalubia Governorate, Egypt. J. Egypt. Soc. Parasitol..

[176] Shaapan R.M., Khalil F.A.M., Abou El Ezz M.T. (2011). Cryptosporidiosis and Toxoplasmosis in native quails of Egypt. Res. J. Vet. Sci..

[177] Helmy Y.A., VON Samson-Himmelstjerna G., Nockler K., Zessin K.H. (2015). Frequencies and spatial distributions of Cryptosporidium in livestock animals and children in the Ismailia province of Egypt. Epidemiol. Infect..

[178] Amer S., Zidan S., Adamu H., Ye J., Roellig D., Xiao L., Feng Y. (2013). Prevalence and characterization of *Cryptosporidium* spp. in dairy cattle in Nile River delta provinces, Egypt. Exp. Parasitol..

[179] Mahfouz M.E., Mira N., Amer S. (2014). Prevalence and genotyping of *Cryptosporidium* spp. in farm animals in Egypt. J. Vet. Med. Sci..

[180] Fereig R.M., AbouLaila M.R., Mohamed S.G., Mahmoud H.Y., Ali A.O., Ali A.F., Hilali M., Zaid A., Mohamed A.E., Nishikawa Y. (2016). Serological detection and epidemiology of Neospora caninum and Cryptosporidium parvum antibodies in cattle in southern Egypt. Acta Trop..

[181] Ibrahim M.A., Abdel-Ghany A.E., Abdel-Latef G.K., Abdel-Aziz S.A., Aboelhadid S.M. (2016). Epidemiology and public health significance of Cryptosporidium isolated from cattle, buffaloes, and humans in Egypt. Parasitol. Res..

[182] Helmy Y.A., Krucken J., Nockler K., von Samson-Himmelstjerna G., Zessin K.H. (2013). Molecular epidemiology of Cryptosporidium in livestock animals and humans in the Ismailia province of Egypt. Vet. Parasitol..

[183] Samn K., Samn A., Abou El-Nour M. (2012). A survey of Giardia and Cryptosporidium spp. in Rural and Urban community in North Delta. Egypt N. Y. Sci. J..

[184] Abd El Kader N.M., Blanco M.A., Ali-Tammam M., Abd El Ghaffar Ael R., Osman A., El Sheikh N., Rubio J.M., de Fuentes I. (2012). Detection of Cryptosporidium parvum and Cryptosporidium hominis in human patients in Cairo, Egypt. Parasitol. Res..

[185] Mousa K.M., Abdel-Tawab A.H., Khalil H.H., El-Hussieny N.A. (2010). Diarrhea due to parasites particularly Cryptosporidium parvum in great Cairo, Egypt. J. Egypt. Soc. Parasitol..

[186] El Shazly A.M., Soltan D.M., El-Sheikha H.M., Sadek G.S., Morsy A.T. (2007). Correlation of ELISA copro-antigen and oocysts count to the severity of cryptosporidiosis parvum in children. J. Egypt. Soc. Parasitol..

[187] Allam A.F., Shehab A.Y. (2002). Efficacy of azithromycin, praziquantel and mirazid in treatment of cryptosporidiosis in school children. J. Egypt. Soc. Parasitol..

[188] Soliman N. (1992). Cryptosporidium infection among primary school children in a rural area in Alexandria. J. Egypt. Public Health Assoc..

[189] Hassan S.I., Sabry H., Amer N.M., Shalaby M.A., Mohamed N.A., Gaballah H. (2002). Incidence of cryptosporidiosis in immunodeficient cancer patients in Egypt. J. Egypt. Soc. Parasitol..

[190] Abdel-Maboud A.I., Rossignol J.F., el-Kady M.S., Mostafa M.S., Kabil S.M. (2000). Cryptosporidiosis in Benha, study of some recent modalities in diagnosis and treatment. J. Egypt. Soc. Parasitol..

[191] Mikhail I.A., Hyams K.C., Podgore J.K., Haberberger R.L., Boghdadi A.M., Mansour N.S., Woody J.N. (1989). Microbiologic and clinical study of acute diarrhea in children in Aswan, Egypt. Scand. J. Infect. Dis..

[192] Abdel-Hafeez E.H., Ahmad A.K., Ali B.A., Moslam F.A. (2012). Opportunistic parasites among immunosuppressed children in Minia district, Egypt. Korean J. Parasitol..

[193] El Naggar H.H., Handousa A.E., El Hamshary E.M., El Shazly A.M. (1999). Evaluation of five stains in diagnosing human intestinal coccidiosis. J. Egypt. Soc. Parasitol..

[194] Antonios S.N., Tolba O.A., Othman A.A., Saad M.A. (2010). A preliminary study on the prevalence of parasitic infections in immunocompromised children. J. Egypt. Soc. Parasitol..

[195] Ali M.S., Mahmoud L.A., Abaza B.E., Ramadan M.A. (2000). Intestinal spore-forming protozoa among patients suffering from chronic renal failure. J. Egypt. Soc. Parasitol..

[196] Sadaka H.A., Gaafar M.R., Mady R.F., Hezema N.N. (2015). Evaluation of ImmunoCard STAT test and ELISA versus light microscopy in diagnosis of giardiasis and cryptosporidiosis. Parasitol. Res..

[197] Banisch D.M., El-Badry A., Klinnert J.V., Ignatius R., El-Dib N. (2015). Simultaneous detection of Entamoeba histolytica/dispar, Giardia duodenalis and cryptosporidia by immunochromatographic assay in stool samples from patients living in the Greater Cairo Region, Egypt. World J. Microbiol. Biotechnol..

[198] Thompson R.C., Hopkins R.M., Homan W.L. (2000). Nomenclature and genetic groupings of Giardia infecting mammals. Parasitol. Today.

[199] Thompson R.C. (2004). The zoonotic significance and molecular epidemiology of Giardia and giardiasis. Vet. Parasitol..

[200] Feng Y., Xiao L. (2011). Zoonotic potential and molecular epidemiology of Giardia species and giardiasis. Clin. Microbiol. Rev..

[201] Younas M., Shah S., Talaat A. (2008). Frequency of Giardia lamblia infection in children with recurrent abdominal pain. J. Pak. Med. Assoc..

[202] Pierce K.K., Kirkpatrick B.D. (2009). Update on human infections caused by intestinal protozoa. Curr. Opin. Gastroenterol..

[203] Lasek-Nesselquist E., Welch D.M., Thompson R.C., Steuart R.F., Sogin M.L. (2009). Genetic exchange within and between assemblages of Giardia duodenalis. J. Eukaryot. Microbiol..

[204] Helmy Y.A., Klotz C., Wilking H., Krucken J., Nockler K., Von Samson-Himmelstjerna G., Zessin K.H., Aebischer T. (2014). Epidemiology of Giardia duodenalis infection in ruminant livestock and children in the Ismailia province of Egypt: Insights by genetic characterization. Parasit. Vectors.

[205] Soliman R.H., Fuentes I., Rubio J.M. (2011). Identification of a novel Assemblage B subgenotype and a zoonotic Assemblage C in human isolates of Giardia intestinalis in Egypt. Parasitol. Int..

[206] Abdel-Moein K.A., Saeed H. (2016). The zoonotic potential of Giardia intestinalis assemblage E in rural settings. Parasitol. Res..

[207] Khalafalla R.E. (2011). A survey study on gastrointestinal parasites of stray cats in northern region of Nile delta, Egypt. PLoS ONE.

[208] Ghoneim N.H., Abdel-Moein K.A., Saeed H. (2012). Fish as a possible reservoir for zoonotic Giardia duodenalis assemblages. Parasitol. Res..

[209] Foronda P., Bargues M.D., Abreu-Acosta N., Periago M.V., Valero M.A., Valladares B., Mas-Coma S. (2008). Identification of genotypes of Giardia intestinalis of human isolates in Egypt. Parasitol. Res..

[210] Sabry M.A., Taher E.S., Meabed E.M.H. (2009). Prevalence and genotyping of zoonotic Giardia from fayoum governorate, Egypt. Res. J. Parasitol..

[211] El-Naggar S.M., el-Bahy M.M., Abd Elaziz J., el-Dardiry M.A. (2006). Detection of protozoal parasites in the stools of diarrhoeic patients using different techniques. J. Egypt. Soc. Parasitol..

[212] Baiomy A.M., Mohamed K.A., Ghannam M.A., Shahat S.A., Al-Saadawy A.S. (2010). Opportunistic parasitic infections among immunocompromised Egyptian patients. J. Egypt. Soc. Parasitol..

[213] Mahmud M.A., Chappell C.L., Hossain M.M., Huang D.B., Habib M., DuPont H.L. (2001). Impact of breast-feeding on Giardia lamblia infections in Bilbeis, Egypt. Am. J. Trop. Med. Hyg..

[214] Helmy M.M., Abdel-Fattah H.S., Rashed L. (2009). Real-time PCR/RFLP assay to detect Giardia intestinalis genotypes in human isolates with diarrhea in Egypt. J. Parasitol..

[215] Hussein E.M., Ismail O.A., Mokhtar A.B., Mohamed S.E., Saad R.M. (2017). Nested PCR targeting intergenic spacer (IGS) in genotyping of Giardia duodenalis isolated from symptomatic and asymptomatic infected Egyptian school children. Parasitol. Res..

[216] Ghieth M.A., Kotb M.A., Abu-Sarea E.Y., El-Badry A.A. (2016). Molecular detection of giardiasis among children at Cairo University Pediatrics Hospitals. J. Parasit Dis..

[217] Hussein E.M., Zaki W.M., Ahmed S.A., Almatary A.M., Nemr N.I., Hussein A.M. (2016). Predominance of Giardia lamblia assemblage A among iron deficiency anaemic pre-school Egyptian children. Parasitol. Res.

[218] Fahmy H.M., El-Serougi A.O., El Deeb H.K., Hussein H.M., Abou-Seri H.M., Klotz C., Aebischer T., El Sayed Khalifa Mohamed K. (2015). Giardia duodenalis assemblages in Egyptian children with diarrhea. Eur. J. Clin. Microbiol. Infect. Dis..

[219] Ismail M.A., El-Akkad D.M., Rizk E.M., El-Askary H.M., El-Badry A.A. (2016). Molecular seasonality of Giardia lamblia in a cohort of Egyptian children: A circannual pattern. Parasitol. Res.

[220] El-Tantawy N.L., Taman A.I. (2014). The epidemiology of Giardia intestinalis assemblages A and B among Egyptian children with diarrhea: A PCR-RFLP-based approach. J. Egypt. Parasitol. United.

[221] Jones J.L., Kruszon-Moran D., Wilson M., McQuillan G., Navin T., McAuley J.B. (2001). Toxoplasma gondii infection in the United States: Seroprevalence and risk factors. Am. J. Epidemiol..

[222] Dubey J.P. (2010). Toxoplasma gondii infections in chickens (*Gallus domesticus*): Prevalence, clinical disease, diagnosis and public health significance. Zoonoses Public Health.

[223] Dubey J.P., Bhatia C.R., Lappin M.R., Ferreira L.R., Thorn A., Kwok O.C. (2009). Seroprevalence of Toxoplasma gondii and Bartonella spp. antibodies in cats from Pennsylvania. J. Parasitol..

[224] Montoya J.G., Liesenfeld O. (2004). Toxoplasmosis. Lancet.

[225] Tenter A.M., Heckeroth A.R., Weiss L.M. (2000). Toxoplasma gondii: From animals to humans. Int. J. Parasitol..

[226] Pinard J.A., Leslie N.S., Irvine P.J. (2003). Maternal serologic screening for toxoplasmosis. J. Midwifery Womens Health.

[227] Cantos G.A., Prando M.D., Siqueira M.V., Teixeira R.M. (2000). Toxoplasmosis: Occurrence of antibodies antitoxoplasma gondii and diagnosis. Rev. Assoc. Med. Bras..

[228] Remington J.S., McLeod R., Thulliez P., Desmonts G., Remington J.S., Klein J.O., Wilson C.B., Baker C.J. (2006). Toxoplasmosis. Infectious Diseases of Fetus and Newborn Infant.

[229] Al-Kappany Y.M., Rajendran C., Ferreira L.R., Kwok O.C., Abu-Elwafa S.A., Hilali M., Dubey J.P. (2010). High prevalence of toxoplasmosis in cats from Egypt: Isolation of viable Toxoplasma gondii, tissue distribution, and isolate designation. J. Parasitol..

[230] Rifaat M.A., Arafa M.S., Sadek M.S., Nasr N.T., Azab M.E., Mahmoud W., Khalil M.S. (1976). Toxoplasma infection of stray cats in Egypt. J. Trop. Med. Hyg..

[231] Aboul-Magd L.A., Tawfik M.S., Arafa M.S., el-Ridi A.M. (1988). Toxoplasma infection of cats in Cairo area as revealed by IFAT. J. Egypt. Soc. Parasitol..

[232] Abu-Zakham A.A., el-Shazly A.M., Yossef M.E., Romeia S.A., Handoussa A.E. (1989). The prevalence of Toxoplasma gondii antibodies among cats from Mahalla El-Kobra, Gharbia Governorate. J. Egypt. Soc. Parasitol..

[233] Hassanain M.A., BARAKAT A.M., Elfadaly H.A., Hassanain N.A., Shaapan R.M. (2008). Zoonotic impact of *Toxoplasma gondii* sero-prevalence in naturally infected Egyptian kittens. J. Arab Soc. Med. Res..

[234] Al-Kappany Y.M., Lappin M.R., Kwok O.C., Abu-Elwafa S.A., Hilali M., Dubey J.P. (2011). Seroprevalence of *Toxoplasma gondii* and concurrent *Bartonella* spp., feline immunodeficiency virus, feline leukemia virus, and Dirofilaria immitis infections in Egyptian cats. J. Parasitol..

[235] Ibrahim B.B., Salama M.M., Gawish N.I., Haridy F.M. (1997). Serological and histopathological studies on toxoplasma Gondii among the workers and the slaughtered animals in Tanta Abattoir, Gharbia Governorate. J. Egypt. Soc. Parasitol..

[236] Ibrahim H.M., Huang P., Salem T.A., Talaat R.M., Nasr M.I., Xuan X., Nishikawa Y. (2009). Short report: Prevalence of Neospora caninum and Toxoplasma gondii antibodies in northern Egypt. Am. J. Trop. Med. Hyg..

[237] Barakat A.M.A., Elaziz M.M.A., Fadaly H.A. (2009). Comparative diagnosis of toxoplasmosis in Egyptian small ruminants by indirect hemagglutination assay and ELISA. Glob. Vet..

[238] Younis E.E., Abou-zeid N.Z., Zakaria M., Mahmoud M.R. (2015). Epidemiological studies on toxoplasmosis in small ruminants and equine in Dakahlia governorate, Egypt. Assiut Vet. Med. J..

[239] Ghoneim N.H., Shalaby S.I., Hassanain N.A., Zeedan G.S., Soliman Y.A., Abdalhamed A.M. (2010). Comparative study between serological and molecular methods for diagnosis of toxoplasmosis in women and small ruminants in Egypt. Foodborne Pathog. Dis..

[240] Shaapan R.M., El-Nawawi F.A., Tawfik M.A. (2008). Sensitivity and specificity of various serological tests for the detection of Toxoplasma gondii infection in naturally infected sheep. Vet. Parasitol..

[241] Barakat A.M., Salem L.M., El-Newishy A.M., Shaapan R.M., El-Mahllawy E.K. (2012). Zoonotic chicken toxoplasmosis in some Egyptians governorates. Pak. J. Biol. Sci..

[242] El-Massry A., Mahdy O.A., El-Ghaysh A., Dubey J.P. (2000). Prevalence of Toxoplasma gondii antibodies in sera of turkeys, chickens, and ducks from Egypt. J. Parasitol..

[243] Dubey J.P., Graham D.H., Dahl E., Hilali M., El-Ghaysh A., Sreekumar C., Kwok O.C., Shen S.K., Lehmann T. (2003). Isolation and molecular characterization of Toxoplasma gondii from chickens and ducks from Egypt. Vet. Parasitol..

[244] Deyab A.K., Hassanein R. (2005). Zoonotic toxoplasmosis in chicken. J. Egypt. Soc. Parasitol..

[245] Aboelhadid S.M., Abdel-Ghany A.E., Ibrahim M.A., Mahran H.A. (2013). Seroprevalence of Toxoplasma Gondii infection in chickens and humans in Beni Suef, Egypt. Glob. Vet..

[246] Ibrahim H.M., Abdel-Ghaffar F., Osman G.Y., El-Shourbagy S.H., Nishikawa Y., Khattab R.A. (2016). Prevalence of *Toxoplasma gondii* in Chicken samples from delta of Egypt using ELISA, histopathology and immunohistochemistry. J. Parasit. Dis..

[247] El Deeb H.K., Salah-Eldin H., Khodeer S., Allah A.A. (2012). Prevalence of Toxoplasma gondii infection in antenatal population in Menoufia governorate, Egypt. Acta Trop..

[248] Azab M.E., el-Shenawy S.F., el-Hady H.M., Ahmad M.M. (1993). Comparative study of three tests (indirect haemagglutination, direct agglutination, and indirect immunofluorescence) for detection of antibodies to Toxoplasma gondii in pregnant women. J. Egypt. Soc. Parasitol..

[249] El-Nawawy A., Soliman A.T., el Azzouni O., Amer el S., Karim M.A., Demian S., el Sayed M. (1996). Maternal and neonatal prevalence of toxoplasma and cytomegalovirus (CMV) antibodies and hepatitis-B antigens in an Egyptian rural area. J. Trop. Pediatr..

[250] Attia R.A., el-Zayat M.M., Rizk H., Motawea S. (1995). Toxoplasma IgG. & IgM. antibodies. A case control study. J. Egypt. Soc. Parasitol..

[251] Awadalla H.N., El-Temsahy M.M., Sharaki O.A., El Zawawy L.A. (2008). Validity of IgG avidity enzyme linked immunosorbent assay and polymerase chain reaction for the determination of Toxoplasma infections during pregnancy. PUJ.

[252] Hussein A.H., Ali A.E., Saleh M.H., Nagaty I.M., Rezk A.Y. (2001). Prevalence of toxoplasma infection in Qualyobia governorate, Egypt. J. Egypt. Soc. Parasitol..

[253] Ibrahim I., Salah H., El Sayed H., Mansour H., Eissa A., Wood J., Fathi W., Tobar S., Gur R.C., Gur R.E. (2016). Hepatitis C virus antibody titers associated with cognitive dysfunction in an asymptomatic community-based sample. J. Clin. Exp. Neuropsychol..

[254] Mabrouk M.A., Dahawi H.S. (1991). Toxoplasma antibodies in patients with meningoencephalitis. J. Egypt. Soc. Parasitol..

[255] Elsheikha H.M., Azab M.S., Abousamra N.K., Rahbar M.H., Elghannam D.M., Raafat D. (2009). Seroprevalence of and risk factors for Toxoplasma gondii antibodies among asymptomatic blood donors in Egypt. Parasitol. Res..

[256] Kim S.M., Kim Y.I., Pascua P.N., Choi Y.K. (2016). Avian influenza A viruses: Evolution and zoonotic infection. Semin. Respir. Crit. Care Med..

[257] Kuiken T., Fouchier R., Rimmelzwaan G., van den Brand J., van Riel D., Osterhaus A. (2011). Pigs, poultry, and pandemic influenza: How zoonotic pathogens threaten human health. Adv. Exp. Med. Biol..

[258] Abdelwhab E.M., Abdel-Moneim A.S. (2015). Epidemiology, ecology and gene pool of influenza A virus in Egypt: Will Egypt be the epicentre of the next influenza pandemic?. Virulence.

[259] Abdelwhab E.M., Hafez H.M. (2011). An overview of the epidemic of highly pathogenic H5N1 avian influenza virus in Egypt: Epidemiology and control challenges. Epidemiol. Infect..

[260] Arafa A.S., Yamada S., Imai M., Watanabe T., Yamayoshi S., Iwatsuki-Horimoto K., Kiso M., Sakai-Tagawa Y., Ito M., Imamura T. (2016). Risk assessment of recent Egyptian H5N1 influenza viruses. Sci. Rep..

[261] Abdelwhab E.M., Hassan M.K., Abdel-Moneim A.S., Naguib M.M., Mostafa A., Hussein I.T., Arafa A., Erfan A.M., Kilany W.H., Agour M.G. (2016). Introduction and enzootic of A/H5N1 in Egypt: Virus evolution, pathogenicity and vaccine efficacy ten years on. Infect. Genet. Evol..

[262] Afifi M.A., El-Kady M.F., Zoelfakar S.A., Abdel-Moneim A.S. (2013). Serological surveillance reveals widespread influenza A H7 and H9 subtypes among chicken flocks in Egypt. Trop. Anim. Health Prod..

[263] Gomaa M.R., Kandeil A., Kayed A.S., Elabd M.A., Zaki S.A., Abu Zeid D., El Rifay A.S., Mousa A.A., Farag M.M., McKenzie P.P. (2016). Serological evidence of human infection with avian influenza A H7virus in Egyptian poultry growers. PLoS ONE.

[264] Hampson K., Coudeville L., Lembo T., Sambo M., Kieffer A., Attlan M., Barrat J., Blanton J.D., Briggs D.J., Cleaveland S. (2015). Estimating the global burden of endemic canine rabies. PLoS Negl. Trop. Dis..

[265] Schnell M.J., McGettigan J.P., Wirblich C., Papaneri A. (2010). The cell biology of rabies virus: Using stealth to reach the brain. Nat. Rev. Microbiol..

[266] Blancou J., King A.A., Fooks A.R., Aubert M., Wandeler A.I. (2004). Rabies in Europe and the Mediterranean basin: From antiquity to the 19th century. Historical Perspective of Rabies in Europe and the Mediterranean Basin.

[267] Abd El Rahman S. (2015). Detection of Rabies virus and its pathological changes in brain of buffaloes in Egypt. Adv. Anim. Vet. Sci..

[268] Botros B.A., Salib A.W., Mellick P.W., Linn J.M., Soliman A.K., Scott R.M. (1988). Antigenic variation of wild and vaccine rabies strains of Egypt. J. Med. Virol..

[269] (2000). World Survey of Rabies N° 36 for the Year 2000.

[270] Botros B.A., Moch R.W., Kerkor M., Helmy I. (1977). Rabies in the Arab Republic of Egypt: III. Enzootic rabies in wildlife. J. Trop. Med. Hyg..

[271] Botros B.A., Lewis J.C., Kerkor M. (1979). A study to evaluate non-fatal rabies in animals. J. Trop. Med. Hyg..

[272] David D., Hughes G.J., Yakobson B.A., Davidson I., Un H., Aylan O., Kuzmin I.V., Rupprecht C.E. (2007). Identification of novel canine rabies virus clades in the Middle East and North Africa. J. Gen. Virol..

[273] El-Tholoth M., El-Beskawy M., Hamed M.F. (2015). Identification and genetic characterization of rabies virus from Egyptian water buffaloes (*Bubalus bubalis*) bitten by a fox. Virusdisease.

[274] Sinclair J.R., Wallace R.M., Gruszynski K., Freeman M.B., Campbell C., Semple S., Innes K., Slavinski S., Palumbo G., Bair-Brake H. (2015). Rabies in a dog imported from Egypt with a falsified rabies vaccination certificate—Virginia, 2015. MMWR Morb. Mortal. Wkly. Rep..

[275] Matter H., Blancou J., Benelmouffok A., Hammami S., Fassi-Fehri N., King A.A., Fooks A.R., Aubert M., Wandeler A.I. (2004). Rabies in North Africa and Malta. Historical Perspective of Rabies in Europe and the Mediterranean Basin.

[276] Sureau P. (1979). Human rabies in France. Rabies Bull. Eur..

[277] Boshra H., Lorenzo G., Busquets N., Brun A. (2011). Rift valley fever: Recent insights into pathogenesis and prevention. J. Virol..

[278] Breiman R.F., Minjauw B., Sharif S.K., Ithondeka P., Njenga M.K. (2010). Rift Valley Fever: Scientific pathways toward public health prevention and response. Am. J. Trop. Med. Hyg..

[279] Arthur R.R., el-Sharkawy M.S., Cope S.E., Botros B.A., Oun S., Morrill J.C., Shope R.E., Hibbs R.G., Darwish M.A., Imam I.Z. (1993). Recurrence of Rift Valley fever in Egypt. Lancet.

[280] Abd el-Rahim I.H., Abd el-Hakim U., Hussein M. (1999). An epizootic of Rift Valley fever in Egypt in 1997. Rev. Sci. Tech..

[281] Ahmed Kamal S. (2011). Observations on rift valley fever virus and vaccines in Egypt. Virol. J..

[282] Meegan J.M. (1979). The Rift Valley fever epizootic in Egypt 1977–78. 1. Description of the epizzotic and virological studies. Trans. R. Soc. Trop. Med. Hyg..

[283] Bird B.H., Khristova M.L., Rollin P.E., Ksiazek T.G., Nichol S.T. (2007). Complete genome analysis of 33 ecologically and biologically diverse Rift Valley fever virus strains reveals widespread virus movement and low genetic diversity due to recent common ancestry. J. Virol..

[284] Samy A.M., Peterson A.T., Hall M. (2017). Phylogeography of Rift Valley Fever Virus in Africa and the Arabian Peninsula. PLoS Negl. Trop. Dis..

[285] Gad A.M., Feinsod F.M., Allam I.H., Eisa M., Hassan A.N., Soliman B.A., el Said S., Saah A.J. (1986). A possible route for the introduction of Rift Valley fever virus into Egypt during 1977. J. Trop. Med. Hyg..

[286] Drake J.M., Hassan A.N., Beier J.C. (2013). A statistical model of Rift Valley fever activity in Egypt. J. Vector Ecol..

[287] Hoogstraal H., Meegan J.M., Khalil G.M., Adham F.K. (1979). The Rift Valley fever epizootic in Egypt 1977–78. 2. Ecological and entomological studies. Trans. R. Soc. Trop. Med. Hyg..

[288] Youssef B.Z., Donia H.A. (2002). The potential role of rattus rattus in enzootic cycle of Rift Valley Fever in Egypt 2-application of reverse transcriptase polymerase chain reaction (RT-PCR) in blood samples of Rattus rattus. J. Egypt. Public Health Assoc..

[289] Imam I.Z., El-Karamany R., Darwish M.A. (1979). An epidemic of Rift Valley fever in Egypt. 2. Isolation of the virus from animals. Bull. World Health Organ..

[290] El-Bahnasawy M.M., Fadil E.E., Morsy T.A. (2013). Mosquito vectors of infectious diseases: Are they neglected health disaster in Egypt?. J. Egypt. Soc. Parasitol..

[291] Conley A.K., Fuller D.O., Haddad N., Hassan A.N., Gad A.M., Beier J.C. (2014). Modeling the distribution of the West Nile and Rift Valley Fever vector Culex pipiens in arid and semi-arid regions of the Middle East and North Africa. Parasit. Vectors.

[292] Gil H., Qualls W.A., Cosner C., DeAngelis D.L., Hassan A., Gad A.M., Ruan S., Cantrell S.R., Beier J.C. (2016). A model for the coupling of the Greater Bairam and local environmental factors in promoting Rift-Valley Fever epizootics in Egypt. Public Health.

[293] Morsy T.A., el Okbi L.M., Kamal A.M., Ahmed M.M., Boshara E.F. (1990). Mosquitoes of the genus Culex in the Suez Canal Governorates. J. Egypt. Soc. Parasitol..

[294] Turell M.J., Presley S.M., Gad A.M., Cope S.E., Dohm D.J., Morrill J.C., Arthur R.R. (1996). Vector competence of Egyptian mosquitoes for Rift Valley fever virus. Am. J. Trop. Med. Hyg..

[295] Turell M.J., Morrill J.C., Rossi C.A., Gad A.M., Cope S.E., Clements T.L., Arthur R.R., Wasieloski L.P., Dohm D.J., Nash D. (2002). Isolation of west nile and sindbis viruses from mosquitoes collected in the Nile Valley of Egypt during an outbreak of Rift Valley fever. J. Med. Entomol..

[296] Youssef B.Z. (2009). The potential role of pigs in the enzootic cycle of rift valley Fever at alexandria governorate, egyp. J. Egypt. Public Health Assoc..

[297] Mroz C., Gwida M., El-Ashker M., Ziegler U., Homeier-Bachmann T., Eiden M., Groschup M.H. (2017). Rift Valley fever virus infections in Egyptian cattle and their prevention. Transbound. Emerg. Dis..

[298] Imam I.Z., Darwish M.A., El-Karamany R. (1979). An epidemic of Rift Valley fever in Egypt. 1. Diagnosis of Rift Valley fever in man. Bull. World Health Organ..

[299] Corwin A., Habib M., Olson J., Scott D., Ksiazek T., Watts D.M. (1992). The prevalence of arboviral, rickettsial, and Hantaan-like viral antibody among schoolchildren in the Nile river delta of Egypt. Trans. R. Soc. Trop. Med. Hyg..

[300] Darwish M H.H. (1981). Arboviruses infecting humans and lower animals in Egypt: A review of thirty years of research. J. Egypt. Public Health Assoc..

[301] Corwin A., Habib M., Watts D., Darwish M., Olson J., Botros B., Hibbs R., Kleinosky M., Lee H.W., Shope R. (1993). Community-based prevalence profile of arboviral, rickettsial, and Hantaan-like viral antibody in the Nile River Delta of Egypt. Am. J. Trop. Med. Hyg..

[302] Abdel-Wahab K.S., El Baz L.M., El-Tayeb E.M., Omar H., Ossman M.A., Yasin W. (1978). Rift Valley Fever virus infections in Egypt: Pathological and virological findings in man. Trans. R. Soc. Trop. Med. Hyg..

[303] Kilpatrick M.E., Girgis N.I., Farid Z., Sippel J.E. (1991). Epidemiology, prevalence and clinical diagnosis of meningitis at Abbassia Fever Hospital, Cairo, 1966–1989. Trans. R. Soc. Trop. Med. Hyg..

[304] Siam A.L., Meegan J.M., Gharbawi K.F. (1980). Rift Valley fever ocular manifestations: Observations during the 1977 epidemic in Egypt. Br. J. Ophthalmol..

[305] Niklasson B., Meegan J.M., Bengtsson E. (1979). Antibodies to Rift Valley fever virus in Swedish U.N. soldiers in Egypt and the Sinai. Scand. J. Infect. Dis..

[306] Brown J.L., Dominik J.W., Morrissey R.L. (1981). Respiratory infectivity of a recently isolated Egyptian strain of Rift Valley fever virus. Infect. Immun..

[307] Centers for Disease Control and Prevention (1994). Rift Valley fever—Egypt, 1993. MMWR Morb. Mortal. Wkly. Rep..

[308] Abu-Elyazeed R., el-Sharkawy S., Olson J., Botros B., Soliman A., Salib A., Cummings C., Arthur R. (1996). Prevalence of anti-Rift-Valley-fever IgM antibody in abattoir workers in the Nile delta during the 1993 outbreak in Egypt. Bull. World Health Organ..

[309] El E., Nagwa A. (2001). Infection by certain arboviruses among workers potentially at risk of infection. J. Egypt. Public Health Assoc..

[310] Zaki A.M., van Boheemen S., Bestebroer T.M., Osterhaus A.D., Fouchier R.A. (2012). Isolation of a novel coronavirus from a man with pneumonia in Saudi Arabia. N. Engl. J. Med..

[311] Han H.J., Yu H., Yu X.J. (2016). Evidence for zoonotic origins of Middle East respiratory syndrome coronavirus. J. Gen. Virol..

[312] Mohd H.A., Al-Tawfiq J.A., Memish Z.A. (2016). Middle East Respiratory Syndrome Coronavirus (MERS-CoV) origin and animal reservoir. Virol. J..

[313] Badawi A., Ryoo S.G. (2016). Prevalence of comorbidities in the Middle East respiratory syndrome coronavirus (MERS-CoV): A systematic review and meta-analysis. Int. J. Infect. Dis..

[314] Chu D.K., Poon L.L., Gomaa M.M., Shehata M.M., Perera R.A., Abu Zeid D., El Rifay A.S., Siu L.Y., Guan Y., Webby R.J. (2014). MERS coronaviruses in dromedary camels, Egypt. Emerg. Infect. Dis..

[315] Perera R.A., Wang P., Gomaa M.R., El-Shesheny R., Kandeil A., Bagato O., Siu L.Y., Shehata M.M., Kayed A.S., Moatasim Y. (2013). Seroepidemiology for MERS coronavirus using microneutralisation and pseudoparticle virus neutralisation assays reveal a high prevalence of antibody in dromedary camels in Egypt, June 2013. Euro Surveill..

[316] Ali M.A., Shehata M.M., Gomaa M.R., Kandeil A., El-Shesheny R., Kayed A.S., El-Taweel A.N., Atea M., Hassan N., Bagato O. (2017). Systematic, active surveillance for Middle East respiratory syndrome coronavirus in camels in Egypt. Emerg. Microbes. Infect..

[317] Refaey S., Amin M.M., Roguski K., Azziz-Baumgartner E., Uyeki T.M., Labib M., Kandeel A. (2017). Cross-sectional survey and surveillance for influenza viruses and MERS-CoV among Egyptian pilgrims returning from Hajj during 2012–2015. Influenza Other Respir. Viruses.

[318] Food and Agriculture Organization (FAO) MERS-CoV Situation Update. http://www.fao.org/AG/AGAINFO/programmes/en/empres/mers/situation_update.html#2.

[319] Bente D.A., Forrester N.L., Watts D.M., McAuley A.J., Whitehouse C.A., Bray M. (2013). Crimean-Congo hemorrhagic fever: History, epidemiology, pathogenesis, clinical syndrome and genetic diversity. Antivir. Res..

[320] Darwish M.A., Imam I.Z., Omar F.M., Hoogstraal H. (1978). Results of a preliminary seroepidemiological survey for Crimean-Congo hemorrhagic fever virus in Egypt. Acta Virol..

[321] Morrill J.C., Soliman A.K., Imam I.Z., Botros B.A., Moussa M.I., Watts D.M. (1990). Serological evidence of Crimean-Congo haemorrhagic fever viral infection among camels imported into Egypt. J. Trop. Med. Hyg..

[322] Mohamed M., Said A.R., Murad A., Graham R. (2008). A serological survey of Crimean-Congo haemorrhagic fever in animals in the Sharkia Governorate of Egypt. Vet. Ital..

[323] Chisholm K., Dueger E., Fahmy N.T., Samaha H.A., Zayed A., Abdel-Dayem M., Villinski J.T. (2012). Crimean-congo hemorrhagic fever virus in ticks from imported livestock, Egypt. Emerg. Infect. Dis..

[324] Weidmann M., Avsic-Zupanc T., Bino S., Bouloy M., Burt F., Chinikar S., Christova I., Dedushaj I., El-Sanousi A., Elaldi N. (2016). Biosafety standards for working with Crimean-Congo hemorrhagic fever virus. J. Gen. Virol..

[325] McVey D.S., Wilson W.C., Gay C.G. (2015). West Nile virus. Rev. Sci. Tech..

[326] Benjelloun A., El Harrak M., Belkadi B. (2016). West Nile disease epidemiology in North-West Africa: Bibliographical review. Transbound. Emerg. Dis..

[327] David S., Abraham A.M. (2016). Epidemiological and clinical aspects on West Nile virus, a globally emerging pathogen. Infect. Dis..

[328] Conte A., Candeloro L., Ippoliti C., Monaco F., De Massis F., Bruno R., Di Sabatino D., Danzetta M.L., Benjelloun A., Belkadi B. (2015). Spatio-temporal identification of areas suitable for West Nile Disease in the Mediterranean basin and Central Europe. PLoS ONE.

[329] El-Bahnasawy M.M., Khater M.K., Morsy T.A. (2013). The mosquito borne West Nile virus infection: Is it threating to Egypt or a neglected endemic disease?. J. Egypt. Soc. Parasitol..

[330] Soliman A., Mohareb E., Salman D., Saad M., Salama S., Fayez C., Hanafi H., Medhat I., Labib E., Rakha M. (2010). Studies on West Nile virus infection in Egypt. J. Infect. Public Health.

[331] Malkinson M., Banet C. (2002). The role of birds in the ecology of West Nile virus in Europe and Africa. Curr. Top. Microbiol. Immunol..

[332] Melnick J.L., Paul J.R., Riordan J.T., Barnett V.H., Goldblum N., Zabin E. (1951). Isolation from human sera in Egypt of a virus apparently identical to West Nile virus. Proc. Soc. Exp. Biol. Med..

[333] Mohammed Y.S., Gresikova M., Adamyova K., Ragib A.H.E.-D.K. (1970). Studies on arboviruses in Egypt. II. Contribution of arboviruses to the aetiology of undiagnosed fever among children. J. Hyg..

[334] Abdel Wahab K.S. (1970). Arboviruses and central nervous system disorders in Egypt. Acta Virol..

[335] Darwish M.A., Ibrahim A.H. (1975). Prevalence of antibodies to arboviruses in Egypt. Results of a serologic survey among 1113 university students. Am. J. Trop. Med. Hyg..

[336] Mohammed Y.S., Sekeyova M., Gresikova M., el-Dawala K. (1968). Studies on arboviruses in Egypt. I. Hemagglutination-inhibition antibodies against arboviruses in human population of Alexandria and Abyss areas. Indian J. Med. Res..

[337] Darwish M.A., Feinsod F.M., Scott R.M., Ksiazek T.G., Botros B.A., Farrag I.H., el Said S. (1987). Arboviral causes of non-specific fever and myalgia in a fever hospital patient population in Cairo, Egypt. Trans. R. Soc. Trop. Med. Hyg..

[338] Darwish M.A., Buck A., Faris R., Gad A., Moustafa A., El-Khashab T., Omar M., Abdel-Hamid T., Shope R.E. (1994). Antibodies to certain arboviruses in humans from a flooded village in Egypt. J. Egypt. Public Health Assoc..

[339] Kropman E., Bakker L.J., de Sonnaville J.J., Koopmans M.P., Raaphorst J., Carpay J.A. (2012). West Nile virus poliomyelitis after a holiday in Egypt. Ned. Tijdschr. Geneeskd..

[340] Salaheldin A.H., Veits J., Abd El-Hamid H.S., Harder T.C., Devrishov D., Mettenleiter T.C., Hafez H.M., Abdelwhab E.M. (2017). Isolation and genetic characterization of a novel 2.2.1.2a H5N1 virus from a vaccinated meat-turkeys flock in Egypt. Virol. J..

[341] Kandeel A., Deming M., Elkreem E.A., El-Refay S., Afifi S., Abukela M., Earhart K., El-Sayed N., El-Gabay H. (2011). Pandemic (H1N1) 2009 and Hajj Pilgrims who received Predeparture Vaccination, Egypt. Emerg. Infect. Dis..

